# Genome analysis and avirulence gene cloning using a high-density RADseq linkage map of the flax rust fungus, *Melampsora lini*

**DOI:** 10.1186/s12864-016-3011-9

**Published:** 2016-08-22

**Authors:** Claire Anderson, Muhammad Adil Khan, Ann-Maree Catanzariti, Cameron A. Jack, Adnane Nemri, Gregory J. Lawrence, Narayana M. Upadhyaya, Adrienne R. Hardham, Jeffrey G. Ellis, Peter N. Dodds, David A. Jones

**Affiliations:** 1 Research School of Biology, The Australian National University, 134 Linnaeus Way, Acton, ACT 2601 Australia; 2 ANU Bioinformatics Consulting Unit, The John Curtin School of Medical Research, The Australian National University, 131 Garran Road, Acton, ACT 2601 Australia; 3 CSIRO Agriculture, GPO Box 1600, Canberra, ACT 2601 Australia; 4 Current address: ARC Centre of Excellence in Plant Energy Biology, The University of Western Australia, 35 Stirling Highway, Crawley, WA 6009 Australia; 5 Current address: KWS SAAT SE, Grimsehlstraße 31, Einbeck, 37574 Germany

**Keywords:** Avirulence gene, Genetic linkage map, Loss of heterozygosity (LOH), Map-based cloning, *Melampsora lini*, Recombination, Restriction-site associated DNA sequencing (RADseq), Rust fungus, Scaffold anchoring

## Abstract

**Background:**

Rust fungi are an important group of plant pathogens that cause devastating losses in agricultural, silvicultural and natural ecosystems. Plants can be protected from rust disease by resistance genes encoding receptors that trigger a highly effective defence response upon recognition of specific pathogen avirulence proteins. Identifying avirulence genes is crucial for understanding how virulence evolves in the field.

**Results:**

To facilitate avirulence gene cloning in the flax rust fungus, *Melampsora lini*, we constructed a high-density genetic linkage map using single nucleotide polymorphisms detected in restriction site-associated DNA sequencing (RADseq) data. The map comprises 13,412 RADseq markers in 27 linkage groups that together span 5860 cM and contain 2756 recombination bins. The marker sequences were used to anchor 68.9 % of the *M. lini* genome assembly onto the genetic map. The map and anchored assembly were then used to: 1) show that *M. lini* has a high overall meiotic recombination rate, but recombination distribution is uneven and large coldspots exist; 2) show that substantial genome rearrangements have occurred in spontaneous loss-of-avirulence mutants; and 3) identify the *AvrL2* and *AvrM14* avirulence genes by map-based cloning. *AvrM14* is a dual-specificity avirulence gene that encodes a predicted nudix hydrolase. *AvrL2* is located in the region of the *M. lini* genome with the lowest recombination rate and encodes a small, highly-charged proline-rich protein.

**Conclusions:**

The *M. lini* high-density linkage map has greatly advanced our understanding of virulence mechanisms in this pathogen by providing novel insights into genome variability and enabling identification of two new avirulence genes.

**Electronic supplementary material:**

The online version of this article (doi:10.1186/s12864-016-3011-9) contains supplementary material, which is available to authorized users.

## Background

Rust fungi are an important group of plant pathogens that cause devastating diseases in agricultural, silvicultural and natural ecosystems. Throughout history rust epidemics have caused significant losses in cereal production and today they are considered a major threat to global food security due to the recent evolution and spread of the highly-virulent Ug99 strain of the wheat stem rust pathogen (*Puccinia graminis* f. sp. *tritici*) and persistent worldwide losses due to wheat stripe rust disease (caused by *Puccinia striiformis* f. sp. *tritici*) [1, 2]. In addition, many other cultivated plants, including soybean, coffee, poplar and pine, suffer rust diseases that drastically reduce productivity [3–6] and the recent incursion of myrtle rust (caused by *Puccinia psidii*) into Australia threatens native ecosystems and forestry production [7]. Consequently, there is great interest in understanding how these fungal pathogens infect their hosts and how plants can be better protected from rust disease.

The infection of flax (*Linum usitatissimum*) by the flax rust fungus (*Melampsora lini*) has been an important model system for understanding rust biology since the pioneering studies of Harold Flor in the late 1930’s and early 1940’s. Flor determined that the success or failure of pathogen attack is dictated by a ‘gene-for-gene’ interaction between resistance genes in the plant and avirulence genes in the pathogen [8]. When a pathogen expressing an avirulence gene infects a plant expressing a corresponding resistance gene, the plant is able to recognise the presence of the pathogen and trigger a multifaceted defence response, which includes localised cell death at the point of attempted infection [9]. Most disease resistance genes encode cytoplasmic receptor proteins with a nucleotide binding (NB) site and leucine rich repeat (LRR) domain [10, 11], whereas avirulence genes encode small, secreted proteins that are thought to be part of a diverse array of secreted effectors with virulence functions that assist in the establishment and maintenance of pathogen infection [12–15]. Five avirulence genes (*AvrL567*, *AvrM*, *AvrP, AvrP123* and *AvrP4*) have been identified at four different loci in *M. lini* [16–18]. They encode secreted proteins produced in a specialised fungal structure called the haustorium, which is a major site for signal exchange and nutrient acquisition in rust pathogens and other biotrophic fungi [19, 20].

Genome sequencing of plant pathogenic fungi has become an important approach to identify effector genes that might be important during infection. For species with relatively small genomes (~40 Mb or less) and a low percentage of repetitive DNA, high quality reference genome assemblies have been produced with large scaffolds that often represent complete chromosomes from telomere to telomere [21–25]. By contrast, species with larger genomes or a high percentage of repetitive DNA have proved more difficult to assemble (e.g. [26]). Rust fungi have repetitive genomes [27–29] that are amongst the largest known for plant pathogens, with genome sizes estimated by flow cytometry that range from 77 Mb in *Puccinia triticina* to 2.5 Gb in *Uromyces bidentis* [30–32]. In addition, they produce dikaryotic spores that are highly heterozygous [29, 33], which makes genome assembly particularly challenging. The wheat stem rust (89 Mb) and poplar rust (Melampsora larici-populina; 101 Mb) pathogen genome assemblies were produced by Sanger sequencing, yet these still contain 392 and 462 scaffolds, respectively [27]. Rust fungal genome assemblies produced using next-generation sequencing (NGS) technologies are considerably more fragmented [29, 34, 35]. For example, the M. lini genome assembly was produced by Illumina sequencing and contains 21,130 scaffolds, which together span 189 Mb of the predicted ~220 Mb genome. The longest scaffold in the assembly is only 239.7 kb and 50 % of the assembly comprises scaffolds of 31.5 kb or less [28].

One strategy to improve the utility of fragmented genome assemblies is to anchor the scaffolds onto a genetic linkage map. In recent years, NGS technologies have been developed that allow simultaneous identification and scoring of single nucleotide polymorphism (SNP) markers, the two most commonly-used methods being the genotyping-by-sequencing (GBS) technique described by Elshire et al. [36] and restriction-site associated DNA sequencing (RADseq; [37]). GBS and RADseq are reduced representation sequencing technologies that rely on restriction digest of genomic DNA to reduce sequence complexity, allowing the high depth of coverage required for accurate SNP identification to be achieved economically. Using these technologies to sequence mapping families it is possible to produce high-density genetic maps that contain thousands of sequence-characterised markers, which can be used for quantitative trait loci (QTL) analysis, map-based cloning, anchoring genome assemblies and genome-wide analysis of recombination rate [38–44]. In fungi, QTLs linked to temperature ensitivity and melanisation have been identified in Zymoseptoria tritici using RADseq markers [45, 46] and a GBS-based linkage map was used to anchor the Pyrenophora teres f. sp. teres genome assembly [47]. However, these powerful technologies have yet to be applied widely in fungal genetics research.

We used RADseq to identify and map genetic markers in an F_2_ family derived by self-fertilisation of M. lini strain CH5 [48]. The resulting high-density genetic map comprises 27 linkage groups that contain 13,412 RADseq markers and nine of the ten avirulence genes known to segregate in this mapping family. In addition the map includes the I-1 avirulence inhibitor locus, which prevents pathogen detection by flax plants containing the L1, L7, L8, L10 and M1 resistance genes [48, 49]. The RADseq marker sequences were used to anchor 68.9 % of the CH5 M. lini genome assembly onto the genetic map. The genetic map and anchored assembly were then used to examine recombination frequency and distribution across the M. lini genome, show that loss of avirulence in some CH5-derived mutants is associated with large-scale chromosomal duplications and deletions and identify two new avirulence genes (AvrM14 and AvrL2) by map-based cloning.

## Results

### CH5 genetic map

We used RADseq to identify SNP-based markers for construction of a M. lini genetic map. Genomic DNAs extracted from strains C and H, their F1 offspring (CH5) and 77 F_2_ progeny obtained by self-fertilisation of CH5 [48] were digested with PstI or NsiI and used to generate individually barcoded RADseq libraries that were sequenced using 100 bp paired-end Illumina sequencing. After filtering to remove poor quality reads and PCR duplicates, on average 2.1 million PstI and 4.4 million NsiI read pairs per F_2_ individual were retained for further analysis (Additional file 1). The sequence data were used to identify 13,617 genetic markers that were heterozygous in CH5, scored in at least 74 of the 77 F_2_ individuals and segregated in a pattern not significantly different from the 1:2:1 ratio expected of a single-copy, codominant marker (chi-squared test, *p* ≥ 0.01). After filtering to remove potential genotyping errors and low-quality markers (see Additional file 2 for a description of methods), a genetic map was constructed at a logarithm of odds (LOD) score of seven that contained 13,412 genetic markers (Additional file 3) in 27 linkage groups (Additional file 4). The map spanned a total of 5860 cM and contained 2756 recombination bins. The individual linkage groups ranged in size from 16 to 480 cM and contained 72 to 1032 genetic markers (Table 1).

**Table 1:** Linkage groups in the CH5 genetic map

Linkage group	Length (cM)	Number of markers	Number of bins	Largest gap (cM)	Mean gap size (cM)
LG1	479.62	999	196	16.70	2.46
LG2	475.13	1032	242	17.87	1.97
LG3	422.07	883	194	9.46	2.19
LG4	378.83	952	188	13.59	2.03
LG5	373.16	765	166	14.89	2.26
LG6	369.61	882	176	15.41	2.11
LG7	346.04	768	151	16.73	2.31
LG8	295.54	599	132	14.47	2.26
LG9	291.43	725	135	14.55	2.17
LG10	270.71	655	114	16.40	2.40
LG11	257.19	554	114	13.72	2.28
LG12	252.32	520	126	13.29	2.02
LG13	238.96	678	125	15.32	1.93
LG14	229.81	457	99	11.53	2.35
LG15	227.36	659	122	11.09	1.88
LG16	176.71	400	83	14.44	2.16
LG17	159.37	293	78	16.55	2.07
LG18	130.74	265	63	7.99	2.11
LG19	111.63	276	52	11.50	2.19
LG20	100.27	273	53	14.37	1.93
LG21	63.56	199	31	12.62	2.12
LG22	60.07	123	24	10.00	2.61
LG23	48.62	113	27	4.30	1.87
LG24	38.49	94	16	8.79	2.57
LG25	26.81	85	22	5.08	1.28
LG26	20.17	91	15	4.03	1.44
LG27	15.99	72	12	4.03	1.45
TOTAL	5860.21	13,412	2756		

**Fig. 1:**
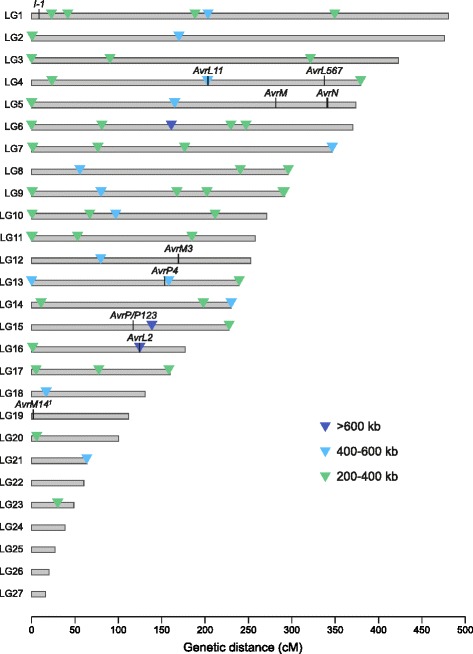
CH5 genetic map. The positions of avirulence genes and the I-1 avirulence inhibitor are indicated; where an avirulence phenotype co-segregates with markers in more than one recombination bin, the map position is indicated using a bracket. Recombination coldspots are indicated by coloured triangles. ^1^ We show in this manuscript that the AvrM1 and AvrM4 avirulence specificities are conferred by a single gene, which we have named AvrM14

Ten avirulence genes segregate in the CH5 F_2_ family and confer 16 avirulence specificities (Additional file 5), with all except AvrN and AvrP inherited from strain H. Nine of these genes were placed on the genetic map along with the I-1avirulence inhibitor (Fig. 1), which prevents pathogen detection by flax plants containing the L1, L7, L8, L10 and M1 resistance genes [48, 49]. Only AvrL1 could not be accurately mapped in this family as it is inhibited by I-1 [48] and there were too few F_2_ individuals for which the AvrL1 genotype could be determined based on the infection phenotype alone (Additional file 5). The avirulence genes and I-1 are distributed throughout the M. lini genome, with no clustering observed. Although AvrL567 and AvrL11 are both located on LG4, they are at least 133.61 cM apart. Similarly, AvrM and AvrN are located at least 58.96 cM apart on LG5. Significantly, co-segregating markers were identified for AvrL2, AvrL11, AvrM1, AvrM3, AvrM4, AvrN and I-1 (Additional file 4), none of which had been cloned.

### Anchoring the CH5 genome assembly

The RADseq markers were used to anchor the M. lini CH5 genome assembly [28] onto the genetic map. In total, 4679 of the 21,310 scaffolds were tagged by RADseq markers, including 1670 (86.6 %) of the 1929 scaffolds in the assembly that were >30 kb in length. The anchored scaffolds had a cumulative length of 130.6 Mb (68.9 % of the total genome assembly length) and contained 11,666 (71.4 %) of the 16,339 [50] predicted M. lini protein coding genes. Physical locations of markers are provided in Additional file 3.

To estimate the error rate in the genetic map, we compared map positions of 1444 pairs of markers derived from either overlapping reads from adjacent restriction sites (220 pairs) or back-to-back reads surrounding a single restriction site (1220 pairs). Disagreement in marker placement was observed in just 22 cases: four were caused by a single missing genotype score; 16 by potential single genotyping errors; and two by both a missing score and a potential genotyping error. By extrapolation, we estimate that just 1.52 % of markers are misplaced by a mean of 0.84 cM (±0.09 cM; standard error). There was also a high level of concordance between the genetic map and the CH5 genome assembly. Of the 739 scaffolds that contained one or more recombinant marker pairs, 687 showed colinearity in physical and genetic marker order allowing unambiguous assignment of scaffold orientation relative to the genetic map (Table 2 and Additional file 6). For another 52 scaffolds, disagreement between physical and genetic marker order may have resulted from single genotyping errors as described above. However, 190 scaffolds showed substantial differences between genetic and physical maps, including 176 that contained markers in more than one linkage group and 15 with widely separated markers from the same linkage group (Table 2 and Additional files 6 and 7). None of these scaffolds contained markers positioned in the correct order to indicate a physical connection between the linkage groups. Therefore, it is likely that these are chimeric scaffolds resulting from errors in sequence assembly.

**Table 2:** Scaffold anchoring summary

Anchoring information	Number of scaffolds
Orientation given by a single recombinant marker pair	490
Orientation given by multiple recombinant marker pairs	197
Genetic order of markers does not match physical order	52
Scaffold chimeric between linkage groups	175
Scaffold chimeric within linkage group	14
Scaffold chimeric between and within linkage groups	1
No recombinant marker pairs on scaffold	1938
<2 markers on scaffold	1812
Total	4679

### Recombination frequency in the M. lini genome

**Fig. 2:**
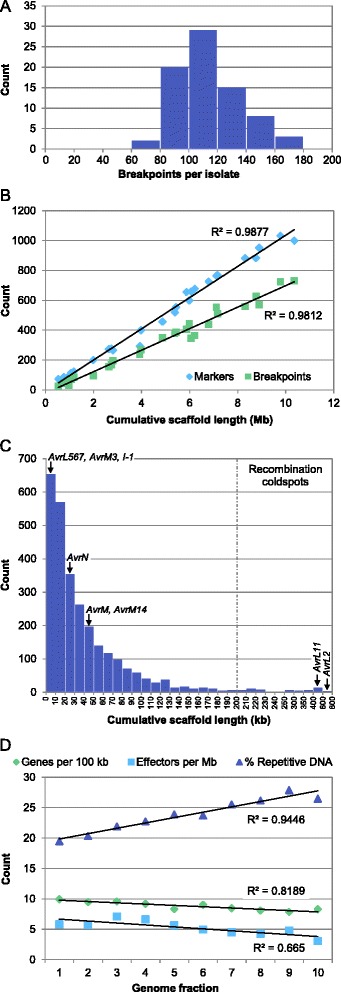
Recombination frequency in the M. lini genome. **a** Histogram of the number of recombination breakpoints per individual in the 77 individuals of the CH5 F_2_ mapping family. **b** Scatter plot showing the correlation between cumulative scaffold length and the number of markers or number of recombination breakpoints per linkage group. Best-fit linear regression lines are shown for each data series. **c** Histogram of the cumulative scaffold length associated with each recombination bin in the CH5 genetic map. Recombination coldspots were considered bins for which the cumulative scaffold length exceeded 200 kb. The cumulative scaffold length associated with bins containing avirulence genes and the I-1 avirulence inhibitor are indicated; where an avirulence gene is associated with more than one bin, the bin with the largest cumulative scaffold length is shown. AvrP/P123 and AvrP4 are not shown as they do not co-segregate with any markers in the CH5 genetic map. See Additional file 2 for a description of how cumulative scaffold length was calculated. **d** DNA sequence content in fractions of the genome with differing recombination rate. The 2756 recombination bins were ordered by increasing cumulative scaffold length and grouped to produce ten genome fractions of approximately equal physical size (11.65 ± 0.01 Mb; see Additional file 9), with group 1 representing regions of the genome with the highest recombination rate and group 10 representing regions of the genome with the lowest recombination rate. Gene density, effector density and the percentage repeat-masked DNA were then determined for each genome fraction. Best-fit linear regression lines are shown for each data series

A total of 8823 recombination events were detected in the 77 members of our F_2_ mapping family. Of these, 8617 (97.7 %) were single recombination events and only 103 locations were detected at which two recombination events had occurred between flanking markers (see Additional file 2 for a description of methods). This suggests that the region of the genome covered by the genetic map is close to marker saturation given the amount of recombination information available in our F_2_ family. The number of recombination breakpoints detected per F_2_ individual ranged from 68 to 166 with a mean of 114.6 (Fig. 2a). Amongst linkage groups, there was a strong positive correlation between physical length and the number of markers (R^2^ = 0.99) and between physical length and the number of recombination events (R^2^ = 0.98; Fig. 2b). However, within linkage groups recombination events were not evenly distributed. Although 50 % of markers were located in bins containing nine markers or less, some bins contained up to 111 markers (Additional file 8) suggesting that there may be recombination coldspots in the genome. To estimate the physical distance between recombination events across the genome, the cumulative scaffold length per recombination bin was calculated (Fig. 2c). Whilst 91.3 % of bins were associated with a cumulative scaffold length of 100 kb or less, there were 59 bins (2.1 %) associated with a physical distance of 200 kb or more (Fig. 1 and Additional file 8). Comparing regions of the genome with differing recombination rates, we found an increase in repetitive DNA content from ~20 to ~26 % as recombination rate decreased, along with a slight decrease in gene and effector gene content (Fig. 2d). There were no differences in GC content between regions with different recombination rates (Additional file 9). Of the mapped M. lini avirulence genes, most were located in recombination bins associated with a physical distance of 50 kb or less (Fig. 2c). The exceptions were AvrL11 and AvrL2, which were located in large recombination coldspots of 477 and 754 kb, respectively.

### Analysis of loss-of-avirulence mutants

**Fig. 3:**
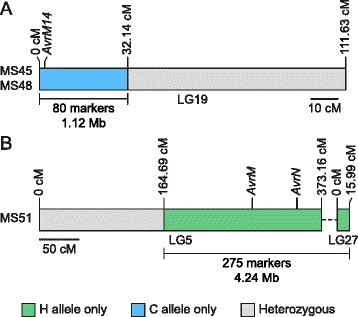
Loss of heterozygosity in CH5 mutants. Regions containing markers for which only the strain H allele is present are shown in green, those for which only the strain C allele is present are shown in blue and those that are heterozygous are shown in grey. The number of markers and cumulative scaffold distance associated with each LOH event are indicated (see Additional file 2 for a description of how cumulative scaffold length was calculated). **a** Genotypes of LG19 markers in MS45 and MS48. AvrM1 and AvrM4 were inherited from strain H and co-segregate in the CH5 F_2_ family [48]. We show later in this paper that they are conferred by a single gene, which we have called AvrM14. **b** Genotypes of LG5 and LG27 markers in MS51. AvrN was inherited from strain C (this study) and AvrM was inherited from strain H [48]. Therefore, MS51 has retained the avirulence allele of AvrM but has lost the avirulence allele of AvrN

As one aim of our research was to identify M. lini avirulence genes, we examined five CH5 mutants that had spontaneously lost either the AvrL2 (MS32, MS38), AvrM1 and AvrM4 (MS45, MS48) or AvrN (MS51) avirulence genes during asexual propagation. Genomic DNAs from these individuals were used for RADseq analysis and the 8871 NsiI markers used in genetic map construction were scored for each line. With the exception of missing data and a small number of potential genotyping errors, all NsiI markers were heterozygous in MS32 and MS38, including those located on LG16 that co-segregated with AvrL2 in the F_2_ family. Therefore, loss of AvrL2 in these mutants must have involved a small-scale mutation event that did not affect the adjacent markers. By contrast, loss of heterozygosity (LOH) was observed for large numbers of markers in MS45, MS48 and MS51 (Fig. 3) suggesting that loss of avirulence in these mutants was caused by large-scale chromosomal rearrangements.

**Fig. 4:**
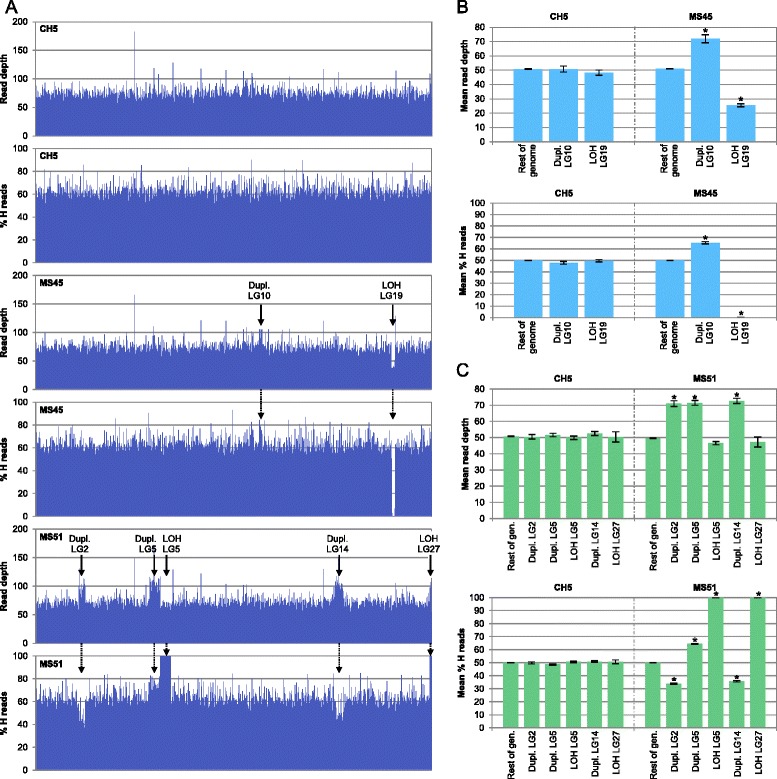
Read depth and percentage of H allele reads in CH5 mutants. **a** Read depth and percentage of H allele reads per marker across the CH5 genetic map. Markers are ordered according to their position in the linkage group. Linkage groups are shown in numerical order, which was assigned according to linkage group size and may not reflect relative position in the genome. Identified regions showing chromosome duplication (Dupl.) or loss of heterozygosity (LOH) are marked by black arrows. Identified regions in MS45 are as follows: Dupl. LG10–64 NsiI markers located between 0 and 10.93 cM; LOH LG19–80 NsiI markers located between 0 and 32.14 cM. Identified regions in MS51 are as follows: Dupl. LG2–155 NsiI markers located between 190.10 and 308.74 cM; Dupl. LG5–261 NsiI markers located between 0 and 164.69 cM; LOH LG5–235 NsiI markers located between 164.69 and 373.16 cM; Dupl. LG14–179 NsiI markers located between 126.41 and 229.81 cM; LOH LG27–40 NsiI markers located between 0 and 15.99 cM. **b** and (**c**) Mean read depth and mean percentage of H allele reads per marker in identified regions compared to the rest of the genome. Significant differences between identified regions and the rest of the genome for a given individual are marked with an asterisk (two-tailed T-test; p < 0.001). Error bars represent ± one standard error

Loss of AvrM1 and AvrM4 in MS45 and MS48 was associated with LOH for 80 consecutive NsiI markers located between 0 and 32.14 cM in LG19 (Fig. 3a). These markers were located on 41 different scaffolds with a cumulative length of 1.12 Mb. To determine the cause of LOH, total read depth and percentage of reads from the strain H allele were examined for all NsiI markers scored in MS45, MS48 and CH5. As shown in Fig. 4a and b, total read depth was uniform across all markers in CH5, but in MS45 the region suffering LOH was characterised by a mean read depth of 25.6 compared to 51.0 for the rest of the genome, consistent with deletion of part of the strain H-derived chromosome that carried AvrM1 and AvrM4. However, this analysis detected a further change in the genome of MS45 that appeared unrelated to loss of AvrM1 and AvrM4; a region of 64 consecutive NsiI markers located between 0 and 10.93 cM in LG10 had higher read depth than the rest of the genome (average of 71.9 reads per marker). These markers were located on 34 scaffolds with a cumulative length of 622 kb. In this region the mean percentage of reads from the strain H allele was 65.4 % compared to 50.0 % for the rest of the genome, consistent with a duplication of this region of the strain H chromosome. Very similar results were obtained for MS48 in both abnormal regions (Additional file 10). As MS45 and MS48 were isolated in the same mutant screen, it is likely that they are clonal individuals.

In MS51, LOH was observed for all 40 NsiI markers in LG27 and for 235 consecutive NsiI markers located between 164.69 cM and the end of LG5 (Fig. 3b). However, in this case avirulence gene loss was not caused by chromosomal deletion alone, as the mean read depth in these two regions was not significantly different to that seen for the rest of the MS51 genome (two-tailed T-test, *p* ≥ 0.001; Fig. 4a, c). Instead, the 261 heterozygous NsiI markers located from 0 to 164.69 cM in LG5 had both increased read depth (71.4 compared to 49.6 for the rest of the genome) and increased percentage of H allele reads (64.5 % compared to 50.1 % for the rest of the genome), suggesting that loss of AvrN was associated with duplication of the strain H chromosome corresponding to LG5 accompanied by deletion of the region containing AvrN from the equivalent chromosome from strain C. A similar chromosome duplication and deletion event could also explain LOH without loss of read depth as observed for LG27 and suggested that LG5 and LG27 might be adjacent regions of the same chromosome. Indeed, when re-analysed at LOD6, markers from these two linkage groups formed a single group separated by a gap of 20.72 cM. Altogether, the combined LG5/LG27 deletion in MS51 affected markers on 172 scaffolds with a cumulative length of 4.24 Mb.

The duplication and deletion events in LG5 and LG27 were not the only large-scale chromosomal changes detected in MS51. Increased read depth was also observed for 155 NsiI markers located between 190.10 and 308.74 cM in the middle of LG2 and for 179 NsiI markers located between 126.41 cM and the end of the linkage group in LG14. In both cases the increased read depth and decreased percentage of H allele reads compared to the rest of the genome was consistent with partial duplication of the chromosomal regions inherited from strain C (Fig. 4a, c), in contrast to the LG5/LG27 duplication event. The duplication in LG2 affected markers on 69 scaffolds with a cumulative scaffold length of 2.48 Mb, whereas the duplication in LG14 affected markers on 131 scaffolds with a cumulative length of 2.64 Mb.

Given the duplication and deletions observed in MS45/48 and MS51, we were interested to know if any of the F_2_ individuals harboured similar chromosomal changes. NsiI marker read depth was examined in all 77 individuals and evidence of H chromosome duplication was observed in CH5F2-133 (Additional file 11). The duplicated region affected 72 consecutive NsiI markers located from 113.68 to 158.65 cM in the middle of LG16. This genetic interval was associated with 53 anchored scaffolds that had a cumulative length of 1.24 Mb. Interestingly the duplicated region contained the largest recombination coldspot in the M. lini genome (LG16; 124.47 cM) and spanned the map location of the AvrL2 avirulence gene.

### Identification of AvrM14

**Fig. 5:**
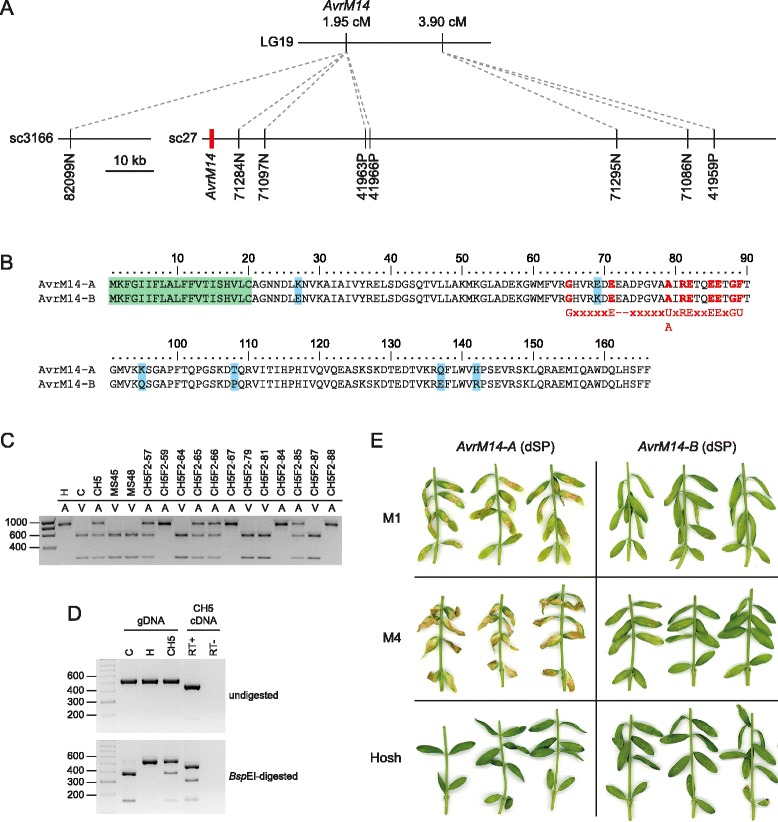
Identification of AvrM14. **a** Physical and genetic maps of the region surrounding AvrM14. The position of AvrM14 is shown in red. **b** Alignment of the predicted AvrM14-A and AvrM14-B protein sequences. The predicted signal peptide is shaded green and polymorphic amino acids are shaded blue. The Nudix box (Gx_5_Ex_5_[U/A]xREx_2_EExGU; [83]) is shown in red, where X represents any amino acid and U represents an aliphatic, hydrophobic amino acid. **c** Genetic mapping of AvrM14 using a CAPS marker. PCR products were amplified from genomic DNAs and then digested with BspEI. The phenotype of each individual on M4 flax plants is indicated as ‘A’ for avirulent or ‘V’ for virulent. Approximate product sizes are shown in base-pairs. **d** RT-PCR analysis of AvrM14 gene expression. Products were amplified from genomic DNA (gDNA) extracted from strain C (virulent on M4 flax), strain H (avirulent on M4 flax) and CH5. Products were also amplified from cDNA made from RNA isolated from flax leaves 4 days post-infection with CH5. RT+ indicates a cDNA reaction that contained reverse transcriptase whereas RT- indicates an identical reaction that lacked reverse transcriptase. Approximate product sizes are shown in base-pairs. **e** Expression of avirulence gene candidates in flax leaves using Agrobacterium tumefaciens-mediated transient transformation. Expression constructs were generated using cDNA sequences that lacked the region encoding the predicted signal peptide (dSP). AvrM14-A is the allele found in strain H, which is avirulent on M1 and M4 flax. AvrM14-B is the allele found in strain C, which is virulent on M1 and M4 flax. Constructs were expressed in flax lines that lacked all known resistance genes (Hoshangabad, shown as ‘Hosh’), or contained only the M1 resistance gene (Williston Brown, shown as ‘M1’) or only the M4 resistance gene (Victory A, shown as ‘M4’). Plants were photographed 8 days after infiltration

Flor [51] showed that AvrM1 and AvrM4 co-segregated in a mapping family that used strain H as the avirulence source and the phenotypic data for the CH5 F_2_ family were consistent with this observation, although the presence of the I-1 inhibitor gene precluded scoring of AvrM1 directly in many F_2_ individuals [48]. We identified five genetic markers located at 1.95 cM in LG19 that co-segregated with AvrM4 in this family (Additional file 4). Four markers were located on scaffold 27 (sc27) in the CH5 genome assembly, which also contained three adjacent markers at 3.9 cM, and the fifth marker was the only marker located on sc3166 (Fig. 5a). These scaffolds were examined for the presence of genes encoding small-secreted proteins, which are generally considered candidates for effectors. However, none of the gene models annotated on the assembly by Nemri et al. [28] fitted these expectations. Therefore, the region was examined manually and a candidate gene was identified at 1588–2336 bp on sc27 that had not been predicted by the annotation pipeline, but was supported by RNAseq transcript assembly data [28] and an incomplete cDNA sequence obtained from the haustorial cDNA library made by Catanzariti et al. [17]. This gene encoded a predicted protein of 166 amino acids with a 20 amino acid predicted signal peptide and showed homology to proteins of the nudix hydrolase superfamily (Nudix_Hydrolase_7 domain CDD: cd04664; e-value 6.63e^−06^).

Sequences corresponding to the avirulence gene candidate were amplified from strain H and strain C genomic DNA. Sequencing revealed seven nucleotide polymorphisms within the coding region (Additional file 12) that resulted in six amino acid polymorphisms between the predicted proteins, none of which were located in conserved residues of the Nudix box motif (Fig. 5b). One of the nucleotide polymorphisms created a BspEI restriction site polymorphism and this was used as the basis of a cleaved amplified polymorphic sequence (CAPS) marker. The CAPS marker co-segregated with AvrM4 in the CH5 F_2_ family and only the strain C (virulence) allele was found in MS45 and MS48 deletion mutants (Fig. 5c). Reverse Transcriptase-Polymerase Chain Reaction (RT-PCR) showed that both alleles of the avirulence gene candidate were expressed in CH5-infected flax leaves 4 days post-infection and confirmed the presence of a 77 bp intron in the coding region (Fig. 5d).

To test for recognition of the candidate avirulence gene by the corresponding M1 and M4 resistance genes, cDNA sequences of the two alleles were used to generate T-DNA constructs for expression in planta. Constructs were produced that contained or lacked the region encoding the predicted signal peptide, as all flax resistance genes cloned to date (including M1) encode predicted cytoplasmic NB-LRR proteins [52] and the interaction between M. lini avirulence proteins and their corresponding NB-LRR proteins occurs inside the plant cell [17, 18]. Agrobacterium tumefaciens-mediated transient expression of the strain H-derived allele without the predicted signal peptide induced strong necrosis in flax plants containing the M4 resistance gene, slightly weaker necrosis in flax plants containing the M1 resistance gene and no symptoms in flax plants lacking these resistance genes, including near-isogenic lines (Fig. 5e and Additional file 13). Therefore, both the AvrM1 and AvrM4 avirulence specificities are conferred by a single gene, which we have named AvrM14-A (GenBank: KU743880) in keeping with the naming convention previously established for AvrL567 and AvrP123 [17, 18]. By contrast, no necrosis or chlorosis was observed on any of the plants infiltrated with the strain C-derived virulence allele, which we have named AvrM14-B (GenBank: KU743881). No response was observed when the full-length AvrM14-A gene was expressed in M1 or M4 plants (Additional file 13).

### Identification of AvrL2

The CH5 genetic map showed that AvrL2 co-segregated with 69 markers at map position 124.47 cM in LG16 (Additional file 4). Co-segregation was observed in 76 of the 77 F_2_ progeny. However, one individual was heterozygous for all markers that co-segregated with AvrL2 in the rest of the F_2_ family, yet was virulent on L2 flax (CH5F2-105; the infection phenotype of which was omitted when placing AvrL2 on the genetic map). This was analogous to the situation seen for the MS32 and MS38 mutants, suggesting that all three individuals might have lost AvrL2 avirulence specificity by a similar process. To identify additional markers that might help to locate AvrL2, we re-analysed the RADseq data using different genotyping calling and filtering parameters that would allow inclusion of multi-copy sequences (Additional file 2). In addition, as approximately 15 % of the RADseq reads did not align to the CH5 genome assembly (Additional file 1), the data were also analysed by de novo assembly. This led to the identification of 1672 additional markers from reference-aligned reads and 6398 markers from de novo assembly, five of which co-segregated with AvrL2 in the 76 F_2_ progeny and were represented by only the strain C allele in CH5F2-105, MS32 and MS38. Three of these markers were located on sc4334 and two were derived from repetitive sequences that could not be placed accurately in the CH5 genome assembly (Additional file 14).

**Fig. 6:**
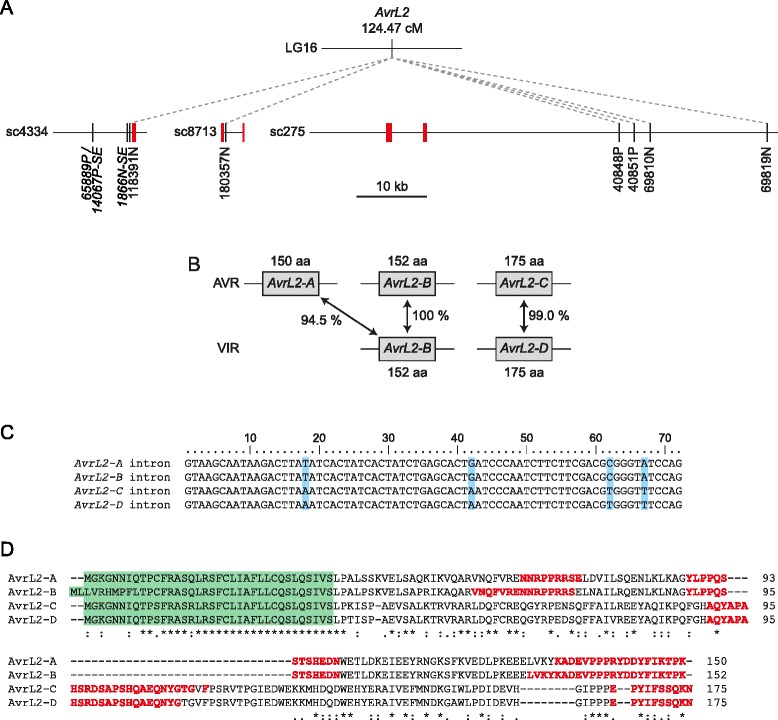
Identification of an AvrL2 gene family. **a** Physical and genetic maps of the region surrounding AvrL2. The positions of AvrL2 family members are shown in red. Genetic markers that were not used in CH5 genetic map construction are shown in italics. **b** AvrL2 family members found in avirulent (AVR) and virulent (VIR) CH5 F_2_ individuals. The percentage DNA identity between coding regions is shown along with the predicted protein size in amino acids (aa). **c** Alignment of introns found in AvrL2 family genes. Polymorphic nucleotides are shaded blue. **d** Alignment of predicted AvrL2 family proteins. Predicted signal peptides are shaded green. Identical amino acids are marked by ‘*’, conservative amino acid substitutions are marked by ‘:’ and semi-conservative amino acid substitutions are marked by ‘.’. Predicted regions of intrinsic disorder are shown in red

Scaffold 4334 contained an annotated gene (Melli_sc4334.2) predicted to encode a small, secreted protein. Two other scaffolds contained closely-related sequences (sc8713 and sc275; Fig. 6a and Additional file 15) and both of these contained markers that co-segregated with AvrL2, suggesting the presence of a small gene family at this location. Because of the sequence duplications and assembly difficulties in this region (Additional files 14 and 15) we used PCR to confirm the sequences of these candidate genes in each haplotype. Three genes (AvrL2-A, -B and -C) were found in homozygous avirulent F_2_ individuals and two genes (AvrL2-B and -D) were found in homozygous virulent F_2_ individuals (Fig. 6b; GenBank: KU743882, KU743883, KU743884, KU743885). The coding regions of AvrL2-A and AvrL2-B are highly related, as are the coding regions of AvrL2-C and AvrL2-D (Fig. 6b). Between AvrL2-A/B and AvrL2-C/D there is much lower identity at the DNA level (≤52.0 %), but all four genes share a conserved 72 bp intron within the coding region (Fig. 6c) and AvrL2-A, -C and -D encode proteins with conserved N-terminal sequences (Fig. 6d). The predicted proteins are 150–175 aa in length with relatively long predicted signal peptides (36–38 aa) as predicted by SignalP v 4.1 [53]. The mature proteins are rich in proline and charged amino acids and contain predicted intrinsically disordered regions (Fig. 6d), but have no homology to other proteins of known function.

**Fig. 7:**
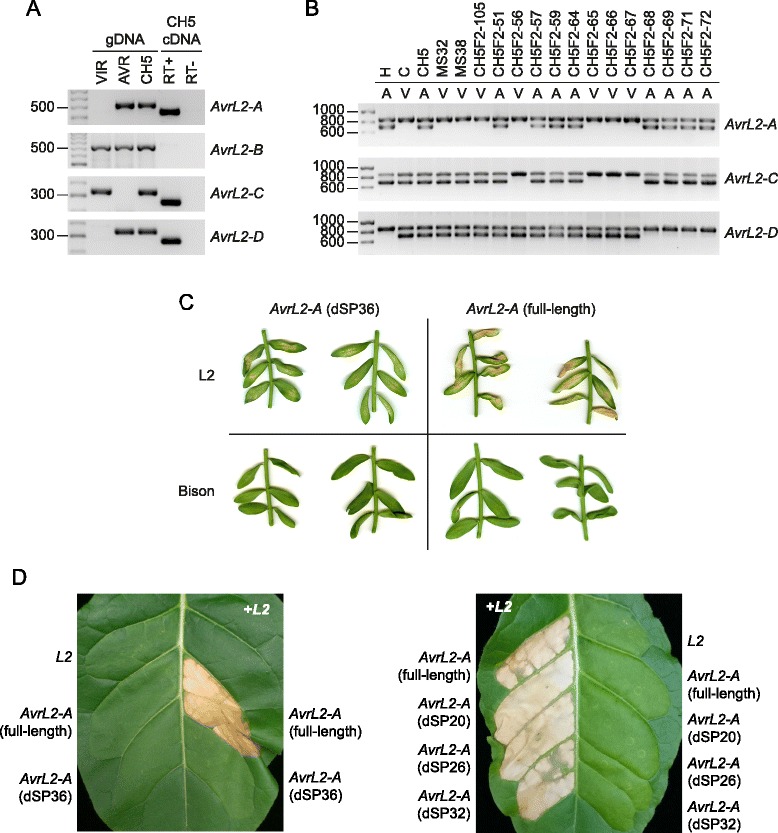
Identification of AvrL2. **a** RT-PCR analysis of AvrL2 family gene expression. Gene-specific primers were used to amplify products by PCR. Products were amplified from genomic DNA (gDNA) extracted from a CH5 F_2_ individual virulent on L2 flax (CH5F2-67, shown as ‘VIR’), a CH5 F_2_ individual avirulent on L2 flax (CH5F2-68, shown as ‘AVR’) and CH5. Products were also amplified from cDNA made from RNA isolated from flax leaves 4 days post-infection with CH5. RT+ indicates a cDNA reaction that contained reverse transcriptase whereas RT- indicates an identical reaction that lacked reverse transcriptase. Approximate product sizes are shown in base-pairs. **b** Genetic mapping of individual AvrL2 family members using gene-specific SCAR markers. Multiplex PCR was performed using primers that amplify an internal control fragment of 861 bp. AvrL2 family members were amplified using gene-specific primers and product sizes are as follows: AvrL2-A, 733 bp; AvrL2-C, 722 bp; and AvrL2-D, 722 bp. The phenotype of each individual on L2 flax plants is indicated as ‘A’ for avirulent or ‘V’ for virulent. Approximate product sizes are shown in base-pairs. **c** Expression of avirulence gene candidates in flax leaves using Agrobacterium tumefaciens-mediated transient transformation. Expression constructs were generated using the full-length AvrL2-A cDNA sequence (full-length) or a sequence that lacked the region encoding the first 36 amino acids (dSP36). Constructs were expressed in near-isogenic flax lines that contained or lacked the L2 resistance gene (B12 × L2, shown as ‘L2’; or Bison, respectively). Plants were photographed 8 days after infiltration. **d** Expression of avirulence gene candidates in Nicotiana tabacum leaves using Agrobacterium tumefaciens-mediated transient transformation. Expression constructs were generated using the full-length AvrL2-A cDNA sequence (full-length) or sequences that lacked the region encoding the first 20, (dSP20), 26 (dSP26), 32 (dSP32) or 36 (dSP36) amino acids. For co-infiltrations, cultures containing AvrL2 and L2 constructs were mixed together in equal ratio. Leaves were photographed 5 days after infiltration

The AvrL2-B coding region was identical in virulent and avirulent individuals, indicating that this gene was not responsible for avirulence on L2 flax. We could not detect expression of AvrL2-B by RT-PCR in either genotype at 4 days post infection (Fig. 7a) and mapping of RNAseq reads from CH5-infected flax (5 days post infection; [28]) did not detect any specific reads corresponding to this gene. However, AvrL2-A, AvrL2-C and AvrL2-D were expressed (Fig. 7a), so we developed sequence characterised amplified region (SCAR) markers specific to each gene and mapped them in the CH5 F_2_ family (Fig. 7b). AvrL2-C and -D segregated as alleles and co-segregated with markers in the recombination bin spanning the AvrL2 locus, but like the other linked markers in this bin they were heterozygous in CH5F2-105, MS32 and MS38. However, AvrL2-A not only co-segregated with the AvrL2 avirulence phenotype in the F_2_ family, but was also absent from CH5F2-105, MS32 and MS38, indicating that it was included in the small deletion event that gave rise to these virulent lines. The full length cDNA sequence of AvrL2-A was confirmed by mapping of RNAseq reads from infected flax [28] onto the genomic sequence and corresponded to a single clone sequenced from a haustorial-specific cDNA library [17]. To test for avirulence function, AvrL2-A expression constructs were generated using cDNA sequences that contained or lacked the region encoding the predicted signal peptide. Initially the signal peptide was predicted using SignalP v4.1 [53], which suggested a signal peptide cleavage site between amino acids 36 and 37 (D-score 0.57). When expressed in flax leaves by Agrobacterium tumefaciens-mediated transient transformation, no symptoms were observed for either construct when expressed in plants that lacked the L2 resistance gene (Fig. 7c and Additional file 13). However, when expressed in L2 flax plants, the AvrL2-A full-length construct induced strong necrosis of infiltrated leaves and the construct lacking the predicted signal peptide-encoding region (dSP36) induced chlorosis and weak necrosis. The weak response to the dSP36 construct was unexpected given that avirulence protein recognition is thought to occur inside the plant cell [17, 18]. However, at 36 amino acids the signal peptide predicted by SignalP v4.1 is unusually long. Therefore, we tested three other constructs that lacked the region encoding the N-terminal 20 (dSP20), 26 (dSP26) or 32 (dSP32) amino acids, the latter corresponding to a signal peptide predicted by PrediSi [54]. When co-expressed with L2 in Nicotiana tabacum, the AvrL2-A full-length, dSP20, dSP26 and dSP32 constructs all elicited a strong necrotic response, whereas no response was observed upon co-expression of AvrL2-A dSP36 and L2 (Fig. 7d). We therefore conclude that AvrL2 avirulence specificity is conferred by the AvrL2-A gene.

## Discussion

We have developed a high-density genetic map for the flax rust fungus (M. lini) that contains 13,412 RADseq markers in 27 linkage groups. As 97.7 % of the 8823 recombination events detected were single recombination events, it is likely that the region of the genome covered by the map is close to marker saturation given the amount of recombination information available in the CH5 F_2_ family. Despite the high marker density, the map contains 27 linkage groups whereas the M. lini haploid genome contains 18 chromosomes [55]. In part, this may be due to the fact we selected markers for map construction that are codominant, predicted single-copy sequences with no strong segregation distortion. Therefore, linkage groups might be broken in regions where markers could not be developed (due to lack of sequence diversity or excessive diversity between the parental strains) or in regions composed entirely of repetitive DNA, provided that sufficient recombination occurred in the region to produce a genetic gap that exceeded the 20 cM threshold used in map construction. Gaps exceeding 20 cM are also expected in regions of strong segregation distortion such as fungal mating type loci, two of which are expected to be heterozygous in every member of the CH5 F_2_ family [56]. Nevertheless, despite M. lini having a repetitive genome [28], at least nine chromosomes are represented by a single linkage group and the high marker density, low level of missing data (0.78 %; Additional file 16) and low estimated marker misplacement rate (1.52 %) makes this genetic map a valuable tool for genomics research.

Genetic maps have been used to anchor a number of fungal genome assemblies, including Fusarium graminearum and Pyrenophora teres f. sp. teres [47, 57, 58]. F. graminearum has a high quality genome assembly [24], 99.8 % of which could be anchored using a genetic map of just 235 markers [57]. By contrast, we were only able to anchor 68.9 % of the M. lini genome assembly onto the genetic map and provide unambiguous orientation to 687 scaffolds. This is due to the fact that the number of genetic markers required to anchor a genome assembly is inversely proportional to the size of sequence scaffolds. Although the RADseq markers are an average of 16.4 kb apart (generating a dense genetic map), 50 % of the assembly is comprised of scaffolds of 31.5 kb or less (a highly fragmented genome assembly), which limits improvement of the anchored assembly.

During the anchoring process we identified 190 scaffolds that contained markers from different linkage groups, or different parts of the same linkage group. The strict alignment parameters used make it unlikely that this is due to incorrect marker placement on the reference genome and none of these markers showed aberrant segregation or high read depth that would indicate they were derived from multi-copy sequences. Instead, the most likely explanation is that the scaffold sequences are chimeric, a common error in assemblies of complex genomes resulting from the inability of sequence assemblers to resolve repetitive DNA stretches from short read sequence data [59, 60]. Scaffolding errors are very difficult to detect by analysis of the assembly alone, prompting some authors to suggest that genetic maps are an essential component of any genome sequencing project [61–63].

Recombination is an essential part of the meiotic cell division process and at least one recombination event (or crossover) is required per chromosome pair to ensure proper segregation of homologous chromosomes during the first meiotic division. However, as aberrant segregation can lead to severe genetic disorders (such as Down syndrome in humans), in most species recombination is tightly controlled to produce a low number of widely spaced recombination events on each chromosome [64]. In Caenorhabditis elegans, Arabidopsis thaliana and many mammalian species, only 1–2 crossover events occur per chromosome pair [65–68]. By contrast, we saw an average of 114.6 recombination breakpoints per F_2_ individual and for the largest linkage group (LG1) up to 22 breakpoints were detected in some F_2_ individuals. Based on the former observation, we infer an average of 6.4 recombination events per chromosome pair during meiosis in M. lini, which is in excess of the number of recombination events thought to be required for correct chromosome disjunction alone. High recombination rates may be typical for fungi as an average of 90.5 recombination events per meiosis have been detected across the 16 chromosome pairs of a diploid Saccharomyces cerevisiae strain [69] and many fungi have relatively large genetic maps given the physical size of their genome. For example, a genetic map of 3006 cM has been constructed for the 90 Mb genome of the pine fusiform rust fungus, Cronartium quercuum f. sp. fusiforme [30, 70]. This is the only other rust fungus for which a comprehensive genetic map currently exists and the size of the map suggests a comparable rate of recombination to that seen here in M. lini. Understanding what controls recombination is an active area of research in many species (see [64, 71]), but we know very little about what controls this process in filamentous fungi. The high rate of fungal recombination is of great benefit in experimental studies as it means that high-resolution mapping can be conducted using relatively small population sizes. In a biological sense, the rapid shuffling of alleles and potential for intragenic recombination (including at avirulence loci) might produce new genetic combinations that are better adapted to changing environmental conditions.

Despite an overall high rate of recombination in the M. lini genome, some regions were devoid of recombination and 59 coldspots were identified in which a recombination bin was associated with a physical region of 200 kb or more (Fig. 2c). In many species strong suppression of recombination is observed at centromeres, which are sites of sister chromatid adhesion during cell division and play a major role in ensuring that chromosomes segregate correctly during mitosis and meiosis [72, 73]. Therefore, some of the coldspots observed in the M. lini genome might correspond to centromeric regions. However, as some linkage groups contained more than one coldspot (Fig. 1), other explanations are possible. Low recombination rates have been associated with non-centromeric heterochromatin and other repeat-rich regions [74, 75] and we observed an increase in mean repetitive DNA content as recombination rate decreased (Fig. 2d). Alternatively, lack of recombination could be caused by chromosomal inversions, translocations or regions of low sequence homology that are specific to the parental combination used to construct the CH5 F_2_ mapping family.

We used genetic markers to examine five mutants that arose spontaneously during asexual propagation of strain CH5 (see [76]) and, in three cases, found that conversion to virulence was associated with large-scale chromosomal alterations. In MS45 and MS48, over 1 Mb of sequence that contained the AvrM14 gene was deleted from the strain H chromosome corresponding to the end of LG19 (Figs. 3a and 4) and a small duplication of the strain H chromosome corresponding to the end of LG10 was also observed in these mutants. The relationship between these two events is currently unknown, but perhaps deletion of LG19 can only be sustained if there is an associated duplication of a function located on LG10. Alternatively, the tip of the LG10 chromosome could have replaced the region deleted from the LG19 chromosome during a translocation event.

MS51 sustained the most substantial and complex chromosomal rearrangements of the mutants examined. In this mutant, loss of AvrN was associated with deletion of over 4 Mb from the strain C chromosome corresponding to LG27 and half of LG5 (Fig. 3b). The deletion in LG5 began in a large recombination coldspot at 164.69 cM that might represent a centromere, suggesting that the deletion may involve loss of a complete chromosome arm. In addition we observed duplication of the entire strain H chromosome corresponding to LG5/LG27 and duplication of strain C sequences corresponding to the central region of LG2 and the end of LG14 (Fig. 4). Again, the relationship between these changes is unclear. However, the fact that deletion of LG5/27 from the strain C-derived nucleus was accompanied by duplication of LG5/27 from the strain H-derived nucleus suggests that there may be physical association or communication between the two haploid nuclei, which have traditionally been considered separate entities in this dikaryotic life stage. Chromosome length polymorphisms and dispensable chromosomes have been observed in many fungal species [77, 78] and loss of heterozygosity has been associated with changes in pathogenicity in the oomycete pathogen, Phytophthora capsici [79], but to our knowledge this is the first demonstration of gross chromosomal changes affecting virulence in a rust fungus. Large-scale chromosomal changes may impose a fitness cost in the field. However, it will be interesting to determine whether such changes contribute to genome evolution in natural populations of rust fungi, particularly for heteroecious species that require two host species to complete their life cycle in regions where reproduction is necessarily asexual due to absence of the sexual stage host.

A major aim of our research was to identify genes controlling avirulence in M. lini and to this end we identified co-segregating markers for six avirulence genes and the I-1 inhibitor gene, none of which had been cloned. In addition, we successfully identified the AvrM14 and AvrL2 genes by map-based cloning. Notably, although recognised by allelic host resistance genes that encode highly homologous proteins [80–82], AvrL2 is unrelated to AvrL567 and similarly AvrM14 is unrelated to AvrM. AvrM14 is a dual specificity avirulence protein that is recognised by both the flax M1 and M4 resistance proteins. Significantly, AvrM14 has homology to proteins of the nudix hydrolase superfamily and is the first rust avirulence protein for which a prediction of biochemical function can be made from the protein sequence alone. Nudix hydrolases are commonly found in both prokaryotes and eukaryotes and are responsible for hydrolysing a wide range of organic pyrophosphates [83]. Several nudix hydrolases have been implicated in plant-pathogen interactions. The Phytophthora sojae Avr3b avirulence protein has proven nudix hydrolase activity [84] and effectors from Colletotrichum truncatum (CtNUDIX; [85]) and Ralstonia solanacearum (Hpx26; [86]) are predicted nudix hydrolase proteins. An Arabidopsis thaliana nudix hydrolase has also been identified that negatively regulates plant defence signalling (NUDT7; [87]). Therefore, secreted pathogen nudix hydrolases might play an important role in manipulating defence activation and cell death responses by the plant, thereby facilitating infection.

AvrL2 is located in the region of the M. lini genome with the lowest recombination rate, which may be a centromere or other heterochromatic or repeat rich region. A number of avirulence genes have been identified in repetitive regions with unique characteristics, such as Leptosphaeria maculans effectors in AT-rich isochores [22] and Phytophthora infestans effectors in gene-sparse genomic regions [88]. The region surrounding AvrL2 shares neither of these characteristics (Additional file 17). However, we found at least two independent mutants in which conversion to virulence was associated with deletion of the AvrL2-A gene and another individual that contained duplication of the AvrL2 locus and surrounding region. This suggests that the AvrL2 locus is prone to rearrangement, reminiscent of L. maculans AvrLm1 and several Magnaporthe oryzae avirulence genes that are located in unstable genomic regions [89–91]. By contrast, virulence alleles at the AvrL567, AvrM, AvrP123 and AvrP4 loci all result from sequence diversification leading to the production of avirulence protein variants that are not recognised by flax plants containing the corresponding resistance genes [92]. Of these cloned avirulence genes, only AvrL2 is located in a recombination coldspot, therefore the genomic context that influences recombination rate may also shape evolution at different M. lini avirulence loci.

The AvrL2 family proteins have no homology to proteins of known function and this is a common feature of pathogen avirulence proteins. However, they do contain several small predicted intrinsically disordered regions, which are known to provide structural flexibility in many proteins involved in cell signalling pathways by allowing them to adopt different conformations in response to post-translational modification and nucleic acid or protein binding [93, 94]. The predicted 114 amino acid, mature AvrL2-A protein contains three small intrinsically disordered regions that together comprise 36 % of the protein sequence (Fig. 6d). Disordered regions have been identified in a number of pathogen effectors. Structural studies have shown that the oomycete Avr3a and Avh5 effectors contain N-terminal disordered regions [95, 96] and the bean rust RTP1p effector contains a predicted N-terminal disordered domain [97]. Moreover, intrinsically disordered regions are enriched in predicted bacterial effectors, leading Marin et al. [98] to propose these may be commonly occurring regions in pathogen effectors that provide the protein flexibility required to enter plant cells, mimic host functions or evade detection by the plant immune system.

## Conclusions

The generation of a high-density genetic map has greatly improved the utility of the fragmented M. lini genome assembly and we have used these genetic resources together to identify two new avirulence genes by map-based cloning. We have also identified RADseq markers that co-segregate with six other avirulence genes and the I-1 inhibitor, which will greatly facilitate future identification of these important genes. The genetic map and anchored assembly have been used to examine recombination rate and frequency across the M. lini genome and the ability to use marker read depth and allele frequency information to calculate DNA copy number has provided novel insights into chromosome rearrangements that occur during asexual reproduction. Taken together, our data suggest that variability in the M. lini genome can be achieved through a high overall recombination rate, consistent with reports of high genetic diversity in natural, sexually reproducing M. lini populations [99], and an ability of the asexual stage to withstand small- and large-scale chromosomal duplications and deletions, which can both impact upon the virulence phenotype. Several mapping families have been produced for other rust fungi, including the wheat stem rust, poplar rust and pine fusiform rust fungi [70, 100, 101]. Approaches like those described here may facilitate genome analysis and avirulence gene cloning in these rust fungi and other species.

## Methods

### Fungal material and DNA extraction

M. lini strains C and H, their F1 hybrid CH5, and an F_2_ family of 77 individuals obtained by self-fertilisation of CH5 have been described previously [48]. Spontaneous M. lini loss-of-avirulence mutants were identified as rare, single uredinial pustules recovered from flax lines containing the L2, M4 or N resistance genes after inoculation with strain CH5 (which is heterozygous for AvrL2, AvrM14 and AvrN). The MS32 and MS38 mutant lines were recovered from L2 flax, MS45 and MS48 were recovered from M4 flax and MS51 was recovered from N flax. All mutant individuals were tested on the full set of flax differential lines and shown only to have gained virulence for the resistance gene present in the host line on which they were recovered (except MS45 and MS48, which were also virulent on M1 flax).

Genomic DNA was extracted from approximately 100 mg of vacuum-dried urediospores according to Nemri et al. [28], except that after isopropanol precipitation the DNA was resuspended in 350 μl of 10 mM Tris–HCl pH 7.4 buffer and further purified with the DNeasy Blood and Tissue kit (Qiagen) using a custom protocol. Samples were mixed with an equal volume of Buffer AL and an equal volume of ethanol before centrifugation through a DNeasy mini spin column at 15,000 g for 1 min. The column was sequentially washed with 500 μl of Buffer AW1, then with 500 μl of Buffer AW2 and dried by centrifugation at 15,000 × g for 3 min. DNA was eluted twice by applying 200 μl Buffer AE to the spin column, incubating at room temperature for 10 min and collecting the eluate by centrifugation at 7000 g for 1 min.

### RADseq library construction, SNP identification and genetic map construction

RADseq libraries were prepared using a protocol adapted from that of Etter et al. [102] and described in full in Additional file 2. In brief, genomic DNA (300 ng) was digested with either PstI (CTGCAG recognition site) or NsiI (ATGCAT recognition site) and ligated to barcoded adapter molecules in a single step reaction. Pooled samples were sonicated and 300–600 bp fragments were isolated by agarose gel electrophoresis and purified. After end-repair and dA-tailing, a second barcoded adapter was ligated to the sheared ends and 400–600 bp size fragments were isolated. The RADseq libraries were amplified before sequencing on an Illumina HiSeq2000 DNA sequencer using 100 bp paired-end sequencing at the Ramaciotti Centre for Genomics, University of New South Wales, Sydney Australia.

RADseq data were analysed using the STACKS v1.04 pipeline [103]. The STACKS genotyping data were converted to MSTmap format and error-checked using a custom Python script (StacksToMSTmap; available from [104]) and the CH5 genetic map was constructed using MSTmap [105]. A set of three custom Python scripts (CHIcheck, DRcheck and BPcheck; available from [104]) were purpose-written to supplement MSTmap and provide map error-checking and analysis functions. A complete description of these processes is provided in Additional file 2.

### Avirulence gene identification and sequence analysis

Candidate avirulence gene sequences were amplified from M. lini genomic DNA by PCR using primers based on the sequences of scaffolds containing co-segregating markers (Additional file 18). PCR products were amplified using Taq polymerase and sequenced directly, or were amplified using Phusion® High-Fidelity DNA polymerase (New England Biolabs), dA-tailed and cloned into pGem®-T Easy (Promega) according to manufacturer’s instructions before Sanger sequencing. Genes were predicted using FGENESH [106] with Puccinia genome-specific parameters. Signal peptides were predicted using SignalP 4.1 [53] (AvrM14 and AvrL2) or PrediSi [54] (AvrL2 only). Similarity searching was performed by BLAST analysis [107] of sequences in the GenBank database and Clustal Omega [108] was used for multiple sequence alignments. Predicted intrinsically disordered regions were identified using the Genesilico MetaDisorder server [109].

### CAPS and SCAR markers

For AvrM14, PCR products were amplified from genomic DNA using primers C34-1 and C34-4 and the products were digested with BspEI. For AvrL2 family members, multiplex PCR was performed using primers C34-1 and C34-4 to amplify an internal control fragment and gene-specific primers to amplify a product that acted as a dominant marker for gene presence. Primer sequences, product sizes and uses can be found in Additional file 18.

### Gene expression analysis

Approximately 3–4-week-old flax (Linum usitatissimum) cv. Hoshangabad plants were inoculated with CH5 urediospores according to Lawrence et al. [48]. Ten infected leaves were collected 4 days post-infection and RNA was extracted using the RNeasy Plant Mini kit (Qiagen) according to manufacturer’s instructions. RNA was treated with DNase I (Promega) before first strand cDNA synthesis using SuperScript® III reverse transcriptase (Life Technologies) essentially according to manufacturer’s instructions. Products were amplified from genomic DNA or cDNA by PCR using primers that flanked introns (Additional file 18).

### In planta transient expression assays

PCR products were amplified from cDNA using primers that would amplify the full coding region, or just the region encoding the predicted mature protein (Additional file 18). In the latter case an artificial ATG codon was inserted in-frame immediately upstream of the avirulence gene sequence to initiate translation. All products were amplified using Phusion® High-Fidelity DNA polymerase (New England Biolabs), dA-tailed and cloned into pGEM®-T Easy (Promega) according to the manufacturer’s instructions. For all AvrM14 constructs, AvrL2-A full-length and AvrL2-A dSP36 the cloned sequences were verified by Sanger sequencing before insertion of the coding region as an EcoRI fragment between the Cauliflower Mosaic Virus (CaMV) 35S promoter and Agrobacterium tumefaciens nopaline synthase terminator sequences in the pTN35S plant expression vector [18]. For AvrL2-A dSP20, dSP26, dSP32 and another version of the full-length AvrL2-A gene, sequences were PCR amplified as described above and cloned into pENTR/D-TOPO (Invitrogen), then transferred into pEarleyGate-102 [110] using LR clonase (Invitrogen). This inserted the sequence between the CaMV 35S promoter and A. tumefaciens octopine synthase terminator of pEarleyGate-102, utilising the natural AvrL2-A stop codon to prevent translation of C-terminal protein tags that are also present in this vector. The L2 expression construct has been described previously [81]. Expression vectors were transformed into A. tumefaciens strain GV3101 (pMP90) and cultures were prepared at an optical density (600 nm) of 1.0 in a buffer containing 10 mM MgCl_2_, 10 mM 2-(N-morpholino) ethanesulphonic acid pH 5.6 and 200 μM acetosyringone for plant infiltration. For co-infiltration experiments, cultures at an optical density (600 nm) of 1.0 were mixed in equal ratio. Cultures were syringe-infiltrated into 3–4 week old flax plants or approximately 8 week old Nicotiana tabacum plants. Infiltrated flax plants were kept in the glasshouse (23 °C by day/13 °C by night, ambient light and relative humidity) for 8–10 days to allow symptoms to develop. At least six leaves on at least three flax plants were infiltrated per experiment and each experiment was repeated at least three times. Infiltrated tobacco plants were kept in a 25 °C growth room with a 16 h day length for 5 days and at least three leaves on either one or two plants were infiltrated per experiment and the experiment was repeated twice.

## Additional files

Additional file 1: RADseq and genetic marker summary. Table showing the numbers of read pairs retained for analysis per CH5 F_2_ individual after pre-processing and removal of PCR duplicates for the NsiI and PstI RADseq sequencing libraries. The numbers of markers of each type used in the CH5 genetic map are also shown. (DOCX 19 kb) Additional file 2: RADseq library construction and computational methods. Detailed methods for RADseq library construction and sequencing, genetic marker identification, genetic map construction and genetic map analysis. (DOCX 41 kb) Additional file 3: Markers used in the CH5 genetic map. Table showing detailed information for all RADseq-derived markers in the CH5 genetic map, including marker sequences. (XLSX 1656 kb) Additional file 4: Linkage groups in the CH5 genetic map. Graphic representations of the 27 linkage groups in the CH5 genetic map, including the positions of the I-1 avirulence inhibitor gene and all mapped avirulence genes. (PDF 6048 kb) Additional file 5: Gene segregation ratios in the CH5 F_2_ mapping family. Segregation ratios of avirulence genes and the I-1 inhibitor gene in the CH5 F_2_ family as obtained by Lawrence et al. [48]. (DOCX 17 kb) Additional file 6: Anchored scaffolds in the M. lini genome assembly. Table showing the numbers of markers, assignment to genetic linkage group and orientation information for each anchored scaffold in the M. lini genome assembly. (XLSX 255 kb) Additional file 7: Within-linkage group chimeric scaffolds. Comparison of physical and genetic maps for scaffolds that are: (A) within-linkage group chimeras; or (B) within- and between-linkage group chimeras. The positions of genetic markers are shown as vertical bars on the physical maps and these are joined to their corresponding positions in the genetic maps by a dotted line. For each gap in the genetic map, the number of markers shown represents those present in recombination bins that lie between the bins containing the markers that flank the chimeric breakpoint. (PDF 838 kb) Additional file 8: Recombination bins in the CH5 genetic map. Table showing the number of markers, number of scaffolds, cumulative scaffold length and sequence characteristics for each recombination bin in the CH5 genetic map. (XLSX 215 kb) Additional file 9: Gene, repeat and nucleotide content in regions of the M. lini genome with different recombination rates. The 2756 recombination bins in the CH5 genetic map were ordered by increasing cumulative scaffold length and grouped to produce ten genome fractions of approximately equal physical size (mean 11,654 kb), with group 1 representing regions of the genome with the highest recombination rate and group 10 representing regions of the genome with the lowest recombination rate. Gene density, effector density and the percentage of GC, N and repeat-masked DNA were then determined for each genome fraction. (DOCX 15 kb) Additional file 10: Loss of heterozygosity in MS48. Graphs showing: (A) read depth and percentage of H allele reads per marker across the CH5 genetic map; and (B) mean read depth and mean percentage of H allele reads per marker in regions of chromosome duplication or deletion in the loss-of-AvrM14 mutant, MS48. (PDF 139 kb) Additional file 11: Chromosome duplication in CH5F2-133. Graphs showing: (A) read depth and percentage of H allele reads per marker across the CH5 genetic map; and (B) mean read depth and mean percentage of H allele reads per marker in a region of chromosome duplication in the CH5 F_2_ individual, CH5F2-133. (PDF 137 kb) Additional file 12: AvrM14 sequences. DNA sequences of AvrM14-A and AvrM14-B and their alignment to sc27. (PDF 377 kb) Additional file 13: Agroinfiltration of avirulence gene constructs. The response of flax cultivars and near-isogenic lines to expression of avirulence gene candidates (AvrM14-A, AvrM14-B and AvrL2-A) using Agrobacterium tumefaciens-mediated transient transformation. (PDF 2637 kb) Additional file 14: RADseq markers that co-segregate with AvrL2 and for which only the strain C allele is present in CH5F2-105, MS32 and MS38. Table showing marker sequences and their scaffold location in the M. lini genome assembly. (DOCX 16 kb) Additional file 15: AvrL2 family DNA sequences. Alignment of AvrL2 coding regions with their closest matching scaffolds in the M. lini genome assembly. Polymorphic nucleotides are shaded. (PDF 337 kb) Additional file 16: Genotyping scores used to make the CH5 genetic map. Table showing the number of markers, number of genotyping scores of each type and number of missing/corrected genotyping scores for each linkage group in the CH5 genetic map. (XLSX 12 kb) Additional file 17: Sequence characteristics in the regions surrounding avirulence genes. Table showing the number of scaffolds, cumulative scaffold length and sequence characteristics of scaffolds that contain and/or contain markers that co-segregate with avirulence genes and the I-1 inhibitor gene. (DOCX 16 kb) Additional file 18: Primers used for PCR amplification of avirulence gene sequences. Table showing primer sequences, amplicon lengths and uses of all primers used to amplify AvrM14 or AvrL2 sequences. (DOCX 20 kb)

## Abbreviations

CaMV: Cauliflower mosaic virus
CAPS: Cleaved amplified polymorphic sequence
GBS: Genotyping by sequencing
LRR: Leucine-rich repeat
LOD: Logarithm of odds
LOH: Loss of heterozygosity
NB: Nucleotide-binding
NGS: Next-generation sequencing
QTL: Quantitative trait loci
RADseq: Restriction site-associated DNA sequencing
SCAR: Sequence characterised amplified region
SNP: Single nucleotide polymorphism
