# Supplementary material for: Genome analysis and avirulence gene cloning using a high-density RADseq linkage map of the flax rust fungus, Melampsora lini
Source: BMC Genomics. 2016 Aug 22;17(1):667. doi: 10.1186/s12864-016-3011-9 (PMC4994203; doi:10.1186/s12864-016-3011-9)
Supplement: Additional file 1: — RADseq and genetic marker summary. Table showing the numbers of read pairs retained for analysis per CH5 F2 individual after pre-processing and removal of PCR duplicates for the NsiI and PstI RADseq sequencing libraries. The numbers of markers of each type used in the CH5 genetic map are also shown. (DOCX 19 kb) [file 12864_2016_3011_MOESM1_ESM.docx]

|  | | ***Nsi*I RADseq data** | ***Pst*I RADseq data** |
| --- | --- | --- | --- |
| Read pairs per CH5 F2 isolate | Mean | 4,430,202 | 2,138,094 |
|  | Coefficient of variation | 10.59 % | 7.07 % |
| Bowtie 2 alignment of RADseq paired end reads to CH5 genome assembly^1^ | No concordant^2^ matches | 28.65 % (± 0.07) | 30.38 % (± 0.05) |
|  | One concordant^2^ match | 41.33 % (± 0.05) | 31.99 % (± 0.03) |
|  | >1 concordant^2^ match | 30.02 % (± 0.04) | 37.64 % (± 0.03) |
|  | Overall alignment rate^3^ | 84.92 % (± 0.08) | 85.18 % (± 0.06) |
| Marker types^4^ used in CH5 linkage map | aa/ab | 1,218 | 588 |
|  | ab/aa | 1,884 | 920 |
|  | aa/bb | 4,444 | 2,367 |
|  | cc/ab | 222 | 114 |
|  | ab/cc | 695 | 365 |
|  | ab/cd | 52 | 29 |
|  | ab/ac | 356 | 158 |
| **Total markers** | | **8,871** | **4,541** |

**Additional file 1. RADseq and genetic marker summary.**

The numbers of read pairs retained for analysis per CH5 F_2_ individual after pre-processing and removal of PCR duplicates are shown for the *Nsi*I and *Pst*I RADseq sequencing libraries. Bowtie 2 [1] was used to align sequence reads to the CH5 reference genome assembly produced by Nemri et al. [2]. The numbers of markers of each type used in the CH5 genetic map are also shown.

^1^ Mean alignment rate for CH5 F_2_ individuals with standard error shown in brackets.

^2^ Concordant matches are those in which the orientation of paired reads is convergent and the reads are separated by a gap within an allowed size range.

^3^ The percentage of all reads that align to the reference, irrespective of whether or not they form concordant read pairs.

^4^ Marker types are shown as xx/yy where xx represents the genotype of strain H and yy represents the genotype of strain C.

**References**

[1] Langmead B, Salzberg SL. Fast gapped-read alignment with Bowtie2. Nat Methods. 2012;9:357-359.

[2] Nemri A, Saunders DGO, Anderson C, Upadhyaya NM, Win J, Lawrence GJ, et al. The genome sequence and effector complement of the flax rust pathogen *Melampsora lini*. Front Plant Sci. 2014;5:98.
