# Supplementary material for: Genome analysis and avirulence gene cloning using a high-density RADseq linkage map of the flax rust fungus, Melampsora lini
Source: BMC Genomics. 2016 Aug 22;17(1):667. doi: 10.1186/s12864-016-3011-9 (PMC4994203; doi:10.1186/s12864-016-3011-9)
Supplement: Additional file 2: — RADseq library construction and computational methods. Detailed methods for RADseq library construction and sequencing, genetic marker identification, genetic map construction and genetic map analysis. (DOCX 41 kb) [file 12864_2016_3011_MOESM2_ESM.docx]

**Additional file 2. RADseq library construction and computational methods.**

**RADseq library construction and sequencing**

RADseq libraries were prepared using a protocol adapted from that described by Etter et al. [1]. Each sample was dual-barcoded with a 5-7 bp inline barcode provided by the P5 (restriction enzyme-cut end) adapter and an 8 bp non-inline barcode provided by the P7 (sheared end) adapter. The P5 adapter sequences were based on those previously described by Grabowski et al. [2], but with an extended set of barcodes. P7 adapters were generated by annealing the P7 adapter universal ddC primer (5’-(P)GATCGGAAGAGCACACGTCTGAACTCCAGTCAC(ddC)-3’, where (P) denotes 5’ phosphorylation and (ddC) denotes dideoxycytidine) with a barcoded P7 adapter index primer (5’-CAAGCAGAAGACGGCATACGAGATxxxxxxxxGTGACTGGAGTTCAGACGTGTGCTCTTCCGATC*T-3’, where xxxxxxxx denotes an 8 bp barcode and ‘*’ denotes a phosphorothioate bond). To make the P5 and P7 adapters, complementary primers at a concentration of 10 µM each were combined in 50 mM NaCl, 10 mM Tris-HCl pH 8.0 buffer and heated to 95 ºC for 5 min before slowly cooling to room temperature (~ 10 min).

Genomic DNAs were digested with restriction enzyme and annealed to P5 adapters in individual restriction-ligation reactions containing 300 ng genomic DNA, 1 x ligase buffer (New England Biolabs), 50 mM NaCl, 500 U T4 DNA ligase (New England Biolabs), 5 U restriction enzyme (*Nsi*I or *Pst*I; New England Biolabs) and either 41.7 nM (*Pst*I) or 75 nM (*Nsi*I) of P5 adapter in a total volume of 30 ul. Reactions were mixed thoroughly and incubated at 22 ºC for 16 hr before heat inactivation of the enzymes at 80 ºC for 20 min. The restriction-ligation reactions were pooled (2 pools of 41-42 samples for the *Pst*I library comprising 77 CH5 F_2_ individuals plus duplicate reactions for strains C, H and CH5 or three pools of 29-30 samples for the *Nsi*I library comprising 77 CH5 F_2_ individuals, five loss-of-avirulence CH5 mutants and duplicate reactions for strains C, H and CH5) and aliquots of DNA were sheared using a BioRuptor® NGS (Diagenode) sonicator. Sheared DNA was separated on a 2% (w/v) agarose, 0.5 x TBE gel and fragments in the size range 300-600 bp were cut from the gel. DNA was purified using the MinElute gel extraction kit (Qiagen) then further cleaned using Agencourt AMPure XP beads (Beckman Coulter, Inc.) according to the manufacturer’s instructions. The size-selected, purified DNA samples (1 ug) were end-repaired, dA-tailed and ligated to P7 adapters using the NEBNext® DNA library prep master mix set for Illumina (New England Biolabs) essentially according to manufacturer’s instructions except that 2 µM of P7 adapter was used during adapter ligation and reaction incubation times were increased to 60 min, 60 min and 90 min for end-repair, dA-tailing and adapter ligation, respectively. The adapter-ligated DNA was separated on a 2% (w/v) agarose, 0.5 x TBE gel and fragments in the size range 400-600 bp were cut from the gel. DNA was purified using the MinElute gel extraction kit (Qiagen) and Agencourt AMPure XP beads (Beckman Coulter, Inc.), as before. Each RADseq library was amplified in a 75 µl reaction containing 60 ng adapter-ligated DNA, 1.25 µM NEBNext Universal primer (New England Biolabs), 1.25 µM P7 reverse primer (5’-CAAGCAGAAGACGGCATACGAGA*T-3’, where ‘*’ denotes a phosphorothioate bond) and 1 x NEBNext high-fidelity PCR master mix (New England Biolabs). Samples were heated to 98 ºC for 30 sec followed by 13 cycles of 98 ºC for 10 sec, 65 ºC for 30 sec and 72 ºC for 30 sec, followed by incubation at 72 ºC for 5 min. The amplified samples were separated on a 2% (w/v) agarose, 0.5 x TBE gel and fragments in the size range 400-600 bp were cut from the gel. DNA was purified using the MinElute gel extraction kit (Qiagen) and Agencourt AMPure XP beads (Beckman Coulter, Inc.), as before. Amplified libraries were normalised to 10 ng/ul in Qiagen Buffer EB containing 0.1% (v/v) TWEEN® 20 (Sigma-Aldrich). Libraries were sequenced (*Pst*I, 1.8 lanes; *Nsi*I, 3.6 lanes) on an Illumina HiSeq 2000 DNA sequencer using 100 bp paired-end sequencing at the Ramaciotti Centre for Genomics, University of New South Wales, Sydney, Australia.

**Genetic marker identification**

Sequences were demultiplexed using the non-inline barcode in the P7 adapter by the Ramaciotti Centre for Genomics using proprietary Illumina software (CASAVA v1.8.2). The first 5 bp of sequence was removed from all R2 reads using Trimmomatic v0.25 [3] (key setting: HEADCROP: 5). RADseq data were then analysed using the STACKS v1.04 pipeline [4]. Initially, sequences were demultiplexed using process_radtags (key settings: -e pstI/nsiI -r -c -q -w 0.04 -s 20 -t 92). The 5, 6 or 7 bp inline barcodes adjacent to the restriction site were detected and removed from R1 reads. Reads were removed if they contained uncalled bases or adapter sequences, lacked the inline barcode or restriction site or where the base quality in a 4 bp sliding window dropped below an average raw phred score of 20. All reads (R1 and R2) were truncated to 92 bp by trimming the 3' end. PCR duplicates were then removed using clone_filter. For strains C, H and CH5 the two individually barcoded sequencing samples were combined to create a single sample with approximately twice the number of sequence reads compared to the F_2_ individuals.

R1 reads were used for *de novo* stack assembly using the STACKS denovo_map.pl script (key settings: -m 3 -M 2 -N 4 -n 0 -t). These settings ensured that a minimum of three identical reads were required to create a stack and, within an individual, stacks could be combined if they contained up to two mismatches. Reads that were not used to initiate stacks could be added to existing stacks if they contained up to four mismatches and up to three stacks could be combined to create a locus. The Removal and Deleveraging algorithms were enabled in ustacks to remove or break up highly repetitive stacks. Single nucleotide polymorphisms were called using the 'snp' model in ustacks with a chi-squared significance level of 0.05 used to call a homozygote or heterozygote. No mismatches were allowed between loci when building the catalogue.

R1 and R2 reads were used together for reference genome-guided stack assembly. First, reads were aligned to the *Melampsora lini* CH5 genome sequence produced by Nemri et al. [5] using Bowtie 2 [6] (key settings: --very-sensitive --end-to-end -I 200 -X 600 --score-min L,0,-0.15 --rdg 10,10 --rfg 10,10). These settings ensured that the entire read must align to the reference genome and the minimum and maximum allowable intervals between paired reads were 200 bp and 600 bp, respectively. To be considered a match, the mismatch score must be greater than -13.2, where each mismatch contributes a score of -2 to -6, scaled according to base quality. High gap-opening and gap-extension penalties were used as the sequence reads were obtained from progeny produced by self-fertilisation of the strain used to create the reference genome sequence. Therefore, gaps were considered unlikely if the read was aligning to its true position in the genome. The Bowtie 2-aligned sequence reads were then used to generate stacks using the STACKS ref_map.pl script (key settings: -n 0 -m 5). A minimum of five reads were required to create a stack and single nucleotide polymorphisms were called using the 'snp' model in ustacks with a chi-squared significance level of 0.05 used to call a homozygote or heterozygote. No mismatches were allowed between loci when building the catalogue.

To obtain markers linked to *AvrL2*, the output files generated by the denovo_map.pl or ref_map.pl scripts were used to rerun the genotypes module in STACKS specifying that all stacks must have a minimum read depth of 5, homozygotes must have a minimum read depth of 10, if the ratio of alleles is more than 1 in 10 the stack is called heterozygous but below 1 in 20 the stack is called homozygous (key settings: -m 5 -t gen -r 1 -c --min_hom_seqs 10). The output files were then manually filtered to retain only those markers that were polymorphic in CH5, scored in at least 74 out of 77 F_2_ individuals and had a major genotype frequency of <90%.

To obtain markers for construction of the CH5 genetic map, the output files generated by ref_map.pl were used to rerun the genotypes module in STACKS specifying that all stacks must have a minimum read depth of 10, if the ratio of alleles is greater than 1 in 7 the stack is called heterozygous, but below 1 in 15 the stack is called homozygous (key settings: -m 10 -t gen -r 1 -c --min_hom_seqs 10 --min_het_seqs 0.14 --max_het_seqs 0.067). The output file was manually filtered to remove markers that were homozygous or had a missing genotype score in CH5 and remove markers that had the same genotype in both strains C and H (as the origin of alleles cannot be conclusively be determined for markers of this type). A custom Python script called StacksToMSTmap v0.2.3 (available from [[7]](https://bitbucket.org/cameronjack/mapcheck)) was then used to identify impossible genotype calls and convert them to missing data and to remove markers that were scored in less than 74 out of 77 F_2_ individuals (key setting: -m 3). The script also converted the genotype data into a format compatible with MSTmap [8] ready for genetic map construction.

**Genetic map construction**

The CH5 genetic map was produced using the MSTmap genetic map construction algorithm developed by Wu et al. [8]. However, as this algorithm does not allow markers to be selected on the basis of a chi-squared test of their segregation ratio and has limited functions for error-checking and manual correction of the resulting genetic map, we developed two custom Python scripts (CHIcheck v0.1.1 and DRcheck v0.2.3; [available](https://bitbucket.org/cameronjack/mapcheck) from [7]) that enabled rapid, iterative correction of the high-density genetic maps made using MSTmap. Due to the ease of use and speed of MSTmap combined with CHIcheck/DRcheck, we were able to produce near-finished maps constructed using different combinations of *p*-values for the chi-squared test and LOD scores for map construction to determine the optimal combination for production of the finished map.

For all maps, first the output from StacksToMSTmap was passed through CHIcheck to remove markers with segregation ratios that differed significantly from the 1:2:1 ratio expected of a single-copy marker segregating in the F_2_ family (key settings: -s 0.25:0.5:0.25, -p from 0.05 to 0.0001). Draft genetic maps were then produced at a logarithm of odds (LOD) score of 6, 7 or 8 using MSTmap (key settings: population type RIL2, distance function kosambi, cut_off_p_value 0.000001 to 0.00000001, no map dist 15, no map size 2, missing threshold 0.04, estimation before clustering no, detect bad data no, objective function COUNT). The draft maps were examined using DRcheck (key settings: --max_dr_gap 2, --max_marker_corrections 2, --min_markers_in_group 2). These settings identified single or dual genotype scores that were flanked on both sides by apparent recombination events. No action was taken if the single/dual genotype calls were heterozygous scores flanked by a region homozygous for the allele inherited from strain H on one side and homozygous for the allele inherited from strain C on the other. However, in all other cases the single/dual scores were considered to be likely genotyping errors or non-crossover gene conversion tracts and were converted to missing data. Markers were removed if they contained more than two corrected genotype scores and linkage groups were removed if they contained less than two markers. After correction by DRcheck, a custom Python script called GAPcheck v0.0.3 (key setting: -g 20; available from [7]) was used to identify all gaps >20 cM. Then the data were passed through CHIcheck, MSTmap, DRcheck and GAPcheck again as described above, except that the missing threshold in MSTmap was increased to 0.07. This process was repeated until no further corrections were made by DRcheck, or up to a maximum of 10 iterations (Table 1).

| **LOD score** | **Chi-squared *p*-value** | **MAPcheck iterations required** | **Total markers** | **Linkage groups ≥ 5 markers** | **Markers in largest linkage group** | **Total gaps >20 cM** | **20-30 cM gaps** | **30-40 cM gaps** | **>40 cM gaps** | **Largest gap (cM)** |
| --- | --- | --- | --- | --- | --- | --- | --- | --- | --- | --- |
| 6 | 0.05 | 7 | 12,678 | 16 | 4,374 | 13 | 5 | 5 | 3 | 57.76 |
| 6 | 0.03 | >10 | 13,067 | 11 | 8,046 | 17 | 6 | 9 | 2 | 48.13 |
| 6 | 0.01 | >10 | 13,404 | 10 | 8,253 | 17 | 6 | 10 | 1 | 41.06 |
| 6 | 0.005 | 7 | 13,474 | 10 | 8,290 | 17 | 5 | 10 | 2 | 47.40 |
| 6 | 0.001 | >10 | 13,547 | 8 | 8,344 | 20 | 8 | 8 | 4 | 63.50 |
| 6 | 0.0005 | >10 | 13,560 | 9 | 6,355 | 18 | 7 | 7 | 4 | 75.18 |
| 6 | 0.0001 | >10 | 13,590 | 8 | 8,366 | 18 | 6 | 9 | 3 | 51.40 |
| 7 | 0.05 | 5 | 12,693 | 30 | 1,229 | 1 | 1 | 0 | 0 | 28.64 |
| 7 | 0.03 | 5 | 13,094 | 29 | 1,242 | 1 | 1 | 0 | 0 | 27.38 |
| 7 | 0.01 | 5 | 13,442 | 26 | 1,281 | 1 | 1 | 0 | 0 | 27.38 |
| 7 | 0.005 | 5 | 13,511 | 25 | 1,290 | 1 | 1 | 0 | 0 | 27.38 |
| 7 | 0.001 | 5 | 13,582 | 25 | 1,290 | 1 | 1 | 0 | 0 | 27.52 |
| 7 | 0.0005 | 5 | 13,595 | 25 | 1,290 | 1 | 1 | 0 | 0 | 27.52 |
| 7 | 0.0001 | 5 | 13,619 | 25 | 1,290 | 1 | 1 | 0 | 0 | 27.52 |
| 8 | 0.05 | 5 | 12,695 | 36 | 943 | 0 | 0 | 0 | 0 | n/a |
| 8 | 0.03 | 5 | 13,098 | 34 | 948 | 0 | 0 | 0 | 0 | n/a |
| 8 | 0.01 | 5 | 13,446 | 33 | 953 | 0 | 0 | 0 | 0 | n/a |
| 8 | 0.005 | 5 | 13,514 | 32 | 962 | 0 | 0 | 0 | 0 | n/a |
| 8 | 0.001 | 5 | 13,587 | 31 | 988 | 0 | 0 | 0 | 0 | n/a |
| 8 | 0.0005 | 5 | 13,601 | 31 | 989 | 0 | 0 | 0 | 0 | n/a |
| 8 | 0.0001 | 5 | 13,626 | 31 | 996 | 0 | 0 | 0 | 0 | n/a |

**Table 1. Comparison of near-finished maps generated using MAPcheck/MSTmap.**

Comparison of the near-finished maps showed that those generated at LOD6 contained fewer linkage groups than the known number of chromosomes in the flax rust genome (*n*=18) [9], were often incomplete even after 10 MAPcheck/MSTmap iterations and were dominated by a single linkage group that contained between 4,374 and 8,366 markers. Moreover, the linkage groups in these maps contained multiple large gaps (>20 cM) that were not consistent between maps built using markers selected at different *p*-values in the chi-squared test. By contrast, all maps built at LOD7 contained more linkage groups than chromosomes, were complete after five MAPcheck/MSTmap iterations and contained a single gap >20 cM that remained consistent regardless of *p*-value used in the chi-squared test. Maps built at LOD7 using *p*-values of 0.001, 0.0005 and 0.0001 for the chi-squared test all contained 25 linkage groups. However, as some authors have suggested that markers that show segregation distortion may not map reliably [10], a conservative approach was taken and the near-complete map constructed using LOD7 and a *p*-value of 0.01 was selected for completion. To finish this map, any linkage group with a gap >20 cM was manually split and the ends of each linkage group were repeatedly trimmed to remove any terminal bin that contained a single marker located >1 cM from the adjacent bin. The positions of avirulence genes and the *I-1* avirulence inhibitor were determined using OneMap [11] and graphic representations of the linkage groups were produced using Genetic-mapper v0.5 [12].

**Genetic map analysis**

A custom Python script (BPcheck v0.0.2; [available](https://bitbucket.org/cameronjack/mapcheck) from [7]) was developed to count the number of recombination breakpoints per linkage group per F_2_ individual. Where a region homozygous for one of the parental alleles was adjacent to a heterozygous region, the transition was counted as one recombination breakpoint (*i.e.* a single recombination event). Where a region homozygous for the allele inherited from strain H was directly adjacent to a region homozygous for the allele inherited from strain C, the transition was counted as two breakpoints (*i.e.* a double recombination event; key setting: -z 2), as in our F_2_ family this scenario can only be produced by two recombination events if caused by recombination alone.

A custom Python script was also developed to approximate the physical distance associated with each recombination bin in the map, taking into account the fact that the *M. lini* CH5 genome assembly contains chimeric scaffold sequences (this manuscript) and comprises 21,130 scaffolds with a N50 scaffold length of just 31.5 kb [5]. Where a non-chimeric scaffold contained markers in a single recombination bin, the full length of the scaffold was attributed to the bin. For chimeric scaffolds, 25% of the scaffold length was attributed to each bin associated with markers on that scaffold. For non-chimeric scaffolds that contained markers in more than one recombination bin, the greatest genetic distance between markers on the scaffold was determined (*genetic distance*). The number of ‘virtual bins’ spanned by the scaffold was calculated as (*genetic distance* / 0.65 cM) + 1 (as one recombination event contributes 0.65 cM to the map). The scaffold length was divided equally between the virtual bins and this length was added to the cumulative total for each bin in the genetic map that was associated with markers on the scaffold. The same methodology was used to calculate nucleotide content, repetitive DNA content and gene/effector numbers per bin, with repeat content calculated using the repeat-masked CH5 genome assembly described in [5] and available from [13].

To determine the cumulative scaffold length associated with individual linkage groups and duplications/deletions in avirulence gene mutants, further custom Python scripts were used. The cumulative scaffold length was calculated by summing the total length of all scaffolds that contained markers in the specified region. Where scaffolds were chimeric between linkage groups, the scaffold length attributed to the specified region was 1 / *n* * *scaffold length* (where *n* denotes the number of linkage groups assigned to a given scaffold). For avirulence gene mutants, only two scaffolds in affected regions were within-linkage group chimeras; in both cases the entire genetic region spanned by the scaffold was contained within the deletion/duplication. Therefore, the entire scaffold length was used.

Additional custom Python scripts were developed for scaffold anchoring, genetic map analysis and analysis of loss-of-avirulence CH5 mutants. All custom Python scripts are available on request.

**References**

[1] Etter PD, Bassham S, Hohenlohe PA, Johnson EA, Cresko WA. SNP discovery and genotyping for evolutionary genetics using RAD sequencing. In: Orgogozo V, Rockman MV. Editors. Molecular Methods for Evolutionary Genetics, Methods in Molecular Biology, vol. 772. Springer Science+Business Media, LLC; 2011. p. 157-178.

[2] Grabowski PP, Morris GP, Casler MD, Borevitz JO. Population genomic variation reveals roles of history, adaptation and ploidy in switchgrass. Mol Ecol. 2014;23:4059-4073.

[3] Bolger AM, Lohse M, Usadel B. Trimmomatic: a flexible trimmer for Illumina sequence data. Bioinformatics. 2014;30:2114-2120.

[4] Catchen JM, Amores A, Hohenlohe P, Cresko W, Postlethwait JH. *Stacks*: building and genotyping loci *de novo* from short-read sequences. G3 (Bethesda). 2011; 1:171-182.

[5] Nemri A, Saunders DGO, Anderson C, Upadhyaya NM, Win J, Lawrence GJ, et al. The genome sequence and effector complement of the flax rust pathogen *Melampsora lini*. Front Plant Sci. 2014;5:98.

[6] Langmead B, Salzberg SL. Fast gapped-read alignment with Bowtie2. Nat Methods. 2012;9:357-359.

[7] MAPcheck code repository. <https://bitbucket.org/cameronjack/mapcheck>. Accessed 1 December 2015.

[8] Wu Y, Bhat PR, Close TJ, Lonardi S. Efficient and accurate construction of genetic linkage maps from the minimum spanning tree of a graph. PLoS Genet. 2008;4:e1000212.

[9] Boehm EWA, Bushnell WR. An ultrastructural pachytene karyotype for *Melampsora lini*. Phytopathol. 1992;82:1212-1218.

[10] Cheema J, Dicks J. Computational approaches and software tools for genetic linkage map estimation in plants. Brief Bioinform. 2009;10:595-608.

[11] Margarido GR, Souza AP, Garcia AA. OneMap: software for genetic mapping in outcrossing species. Hereditas. 2007;144:78-79.

[12] Genetic-mapper code repository. https://code.google.com/p/genetic-mapper/. Accessed 28 July 2014.

[13] <http://webapollo.bioinformatics.csiro.au:8080/melampsora_lini/>. Accessed 18 June 2016.
