# Supplementary material for: Genome analysis and avirulence gene cloning using a high-density RADseq linkage map of the flax rust fungus, Melampsora lini
Source: BMC Genomics. 2016 Aug 22;17(1):667. doi: 10.1186/s12864-016-3011-9 (PMC4994203; doi:10.1186/s12864-016-3011-9)
Supplement: Additional file 4: — Linkage groups in the CH5 genetic map. Graphic representations of the 27 linkage groups in the CH5 genetic map, including the positions of the I-1 avirulence inhibitor gene and all mapped avirulence genes. (PDF 6048 kb) [file 12864_2016_3011_MOESM4_ESM.pdf]

Additional file 4. Linkage groups in the CH5 genetic map. The positions of avirulence genes and the *I-1* avirulence inhibitor are indicated; where an avirulence phenotype co-segregates with markers in more than one recombination bin, the map position is indicated using a bracket. All map positions are shown in cM.

[1] We show in this manuscript that the *AvrM1* and *AvrM4* avirulence specificities are conferred by a single gene, which we have named *AvrM14*.

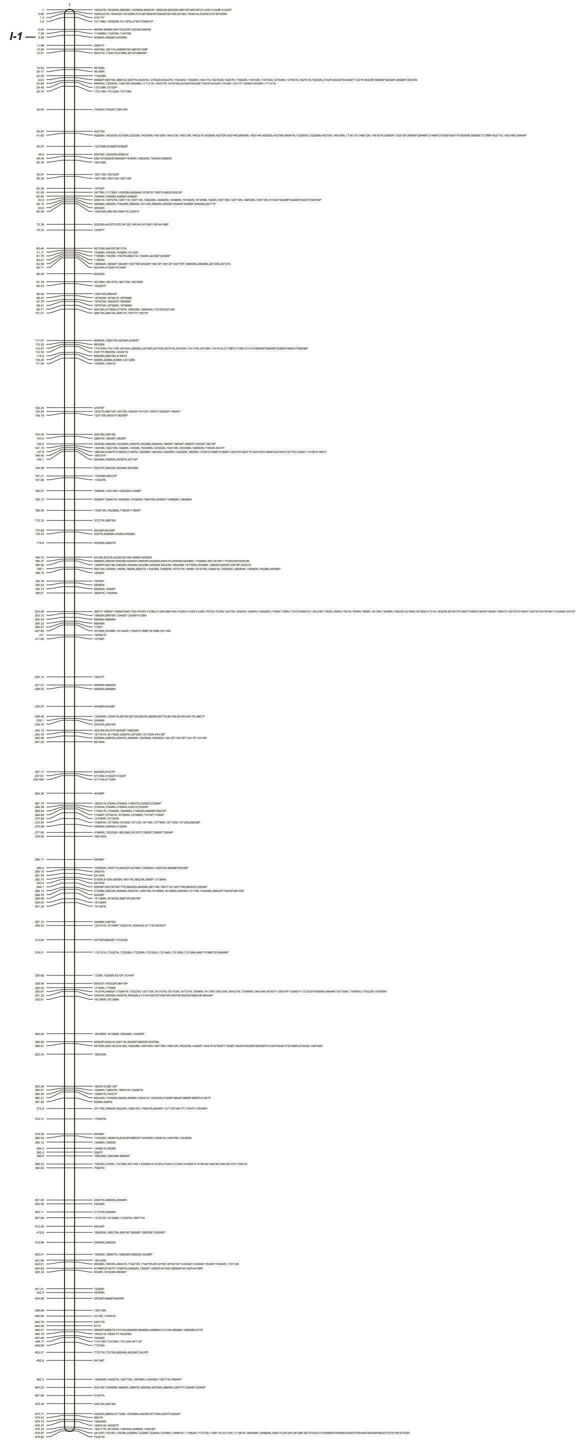

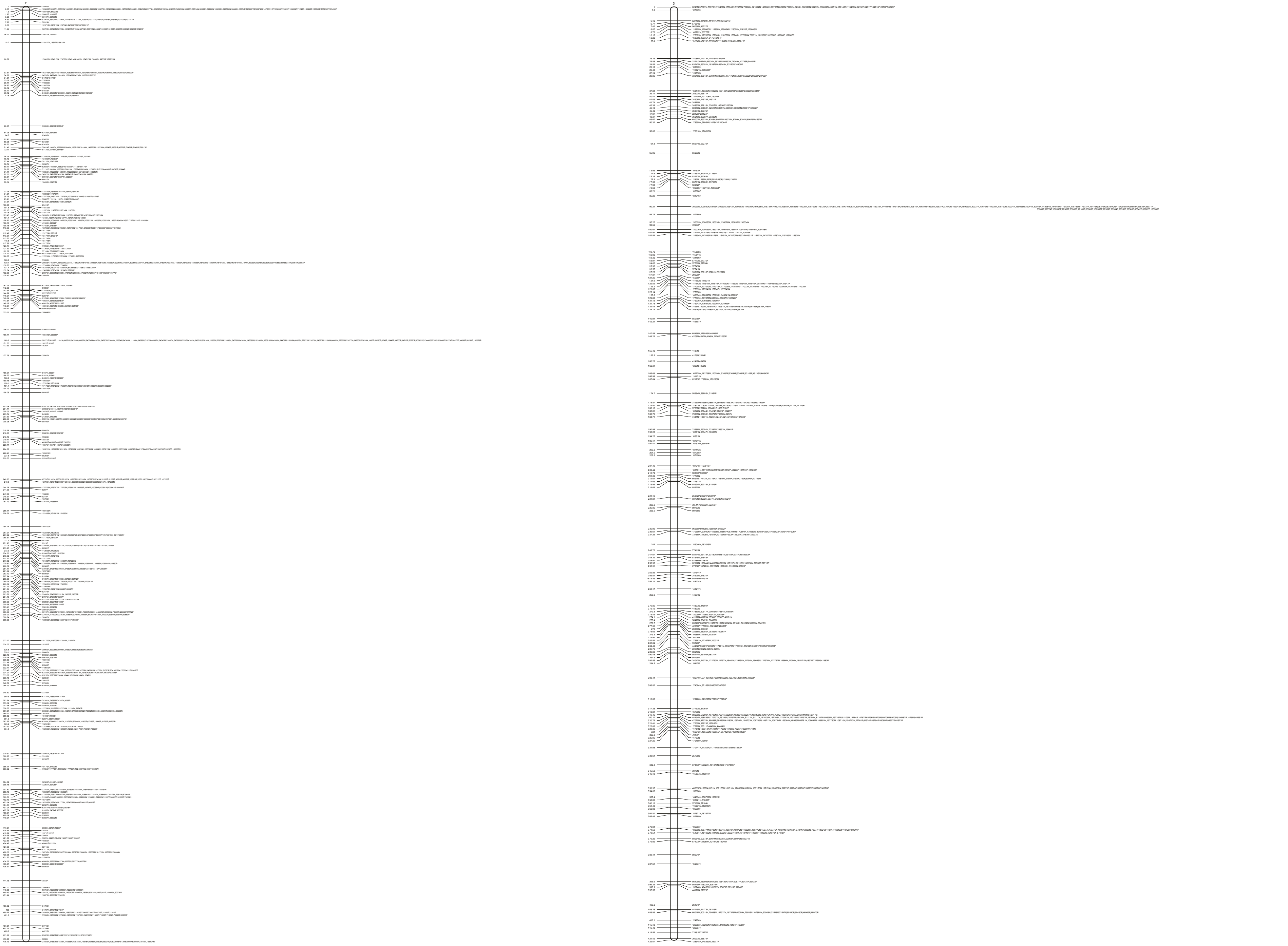
2
3

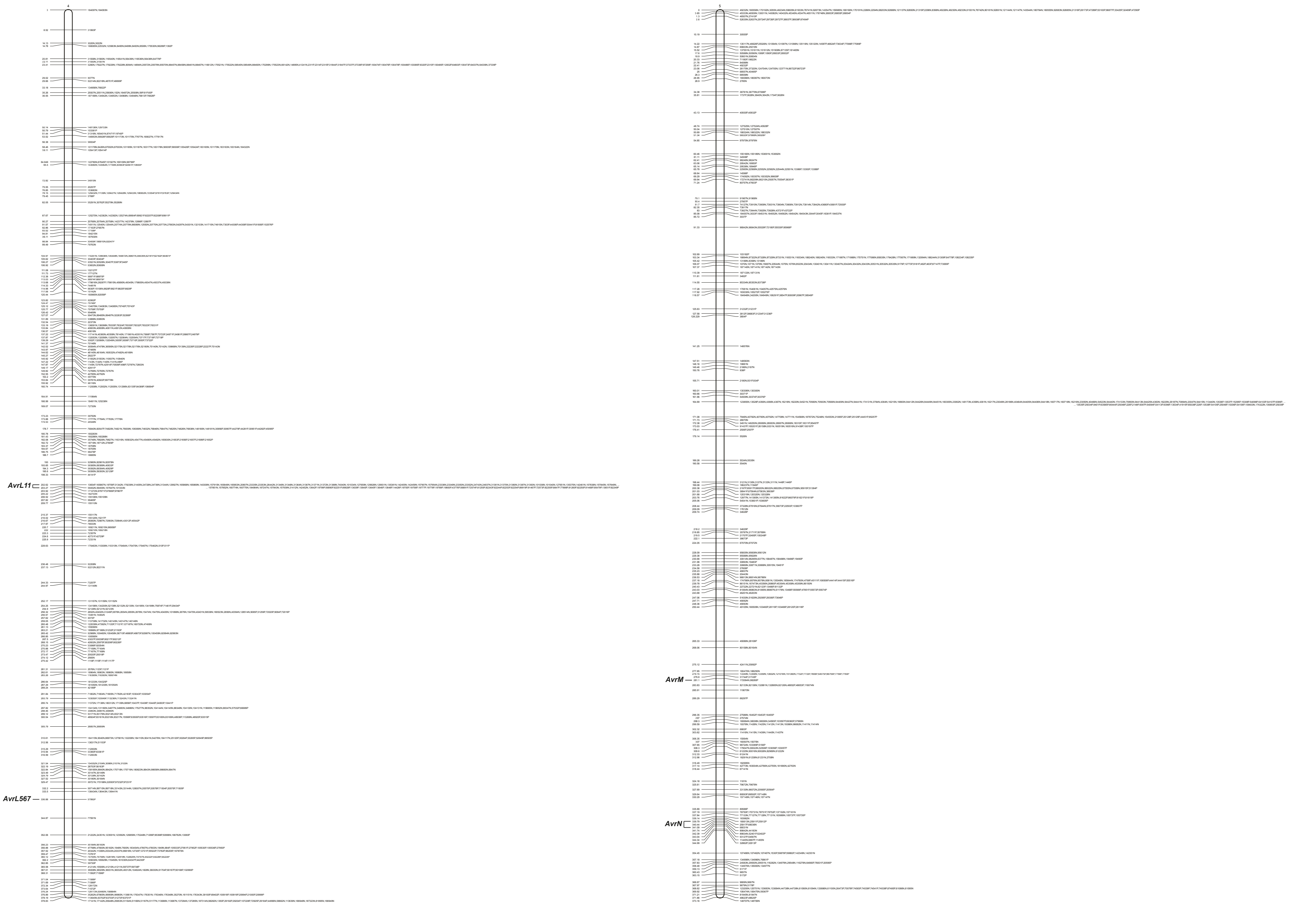
4
5
AvrL11
AvrL567
AvrM
AvrN

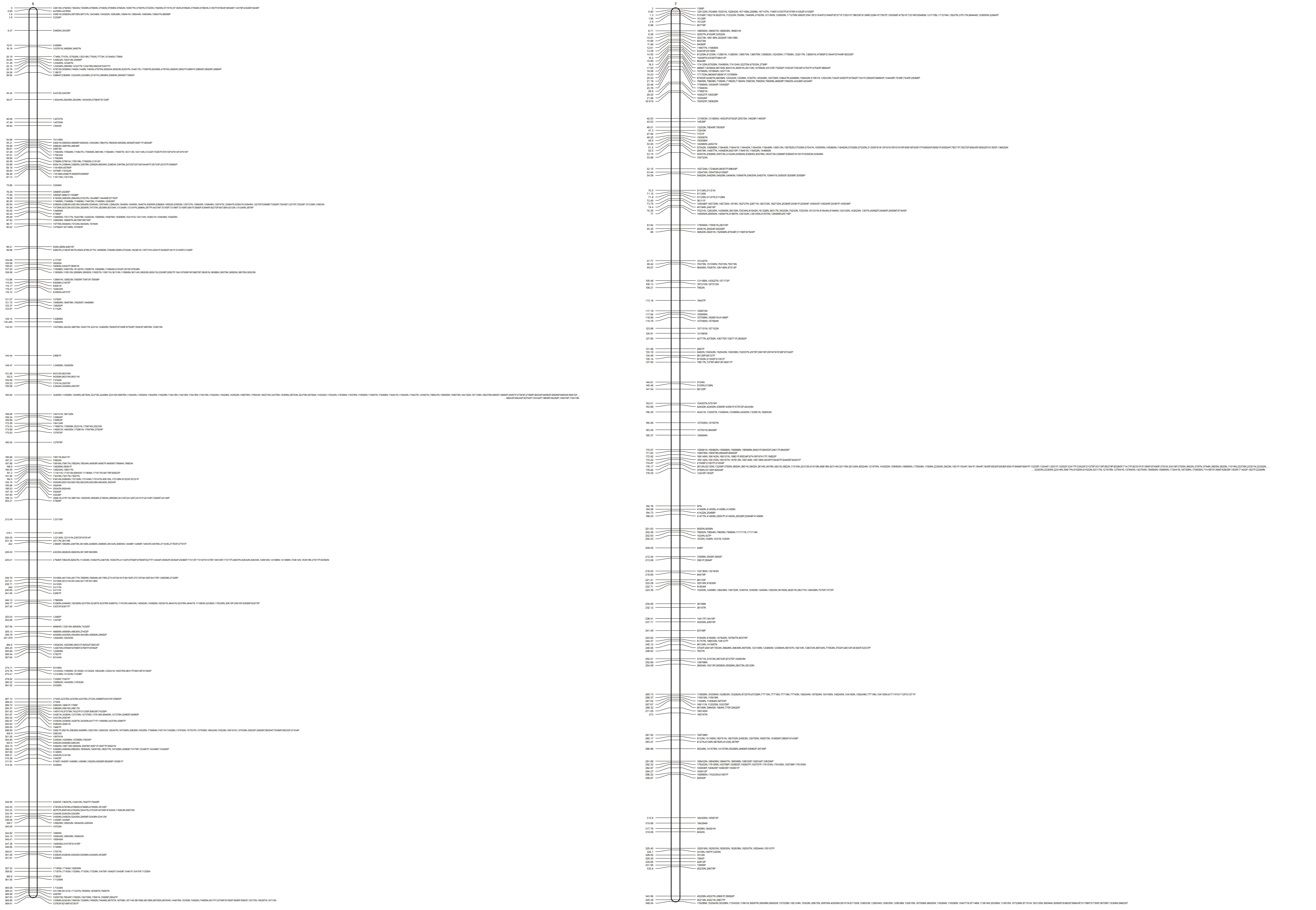
6
7

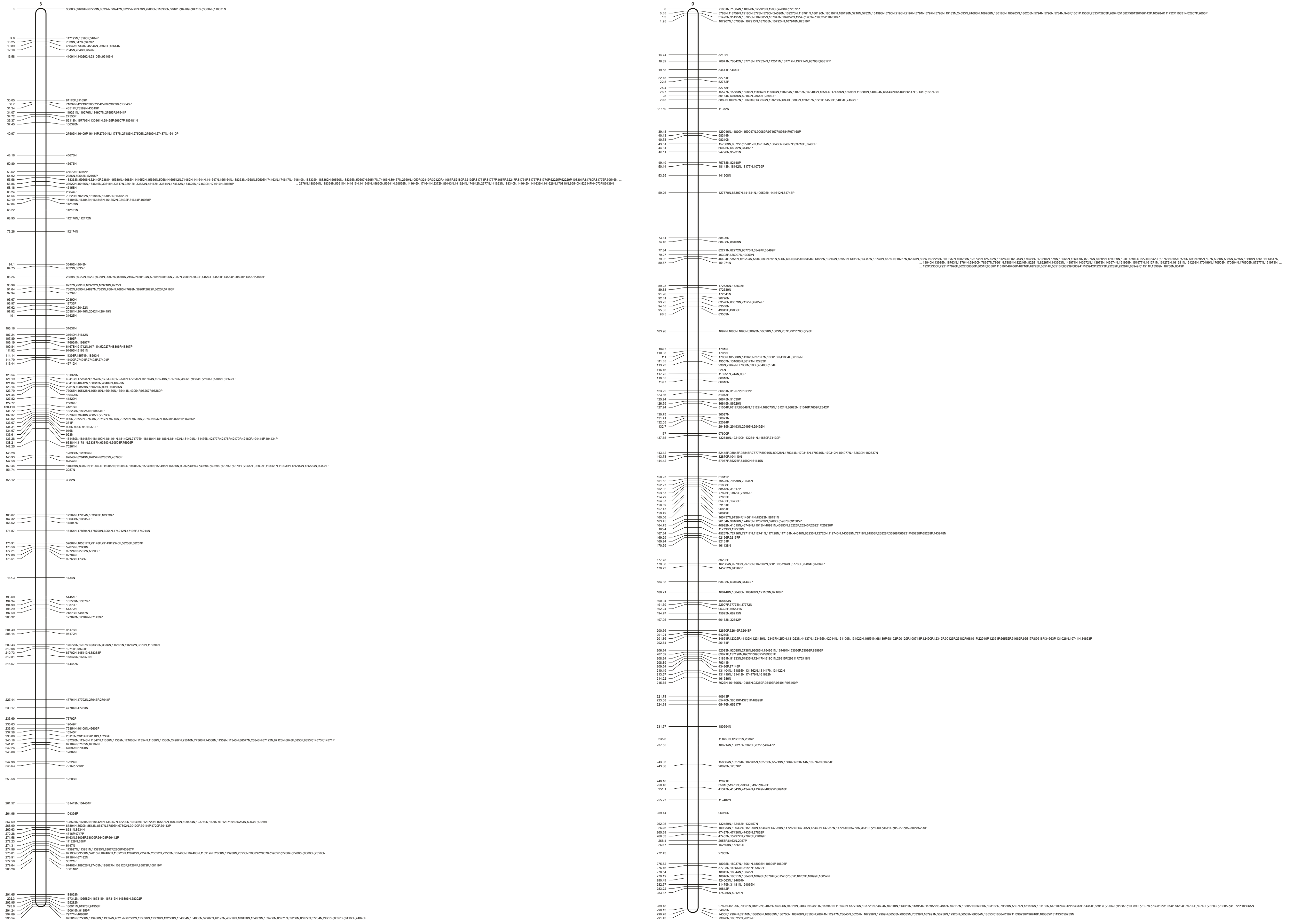

8
9

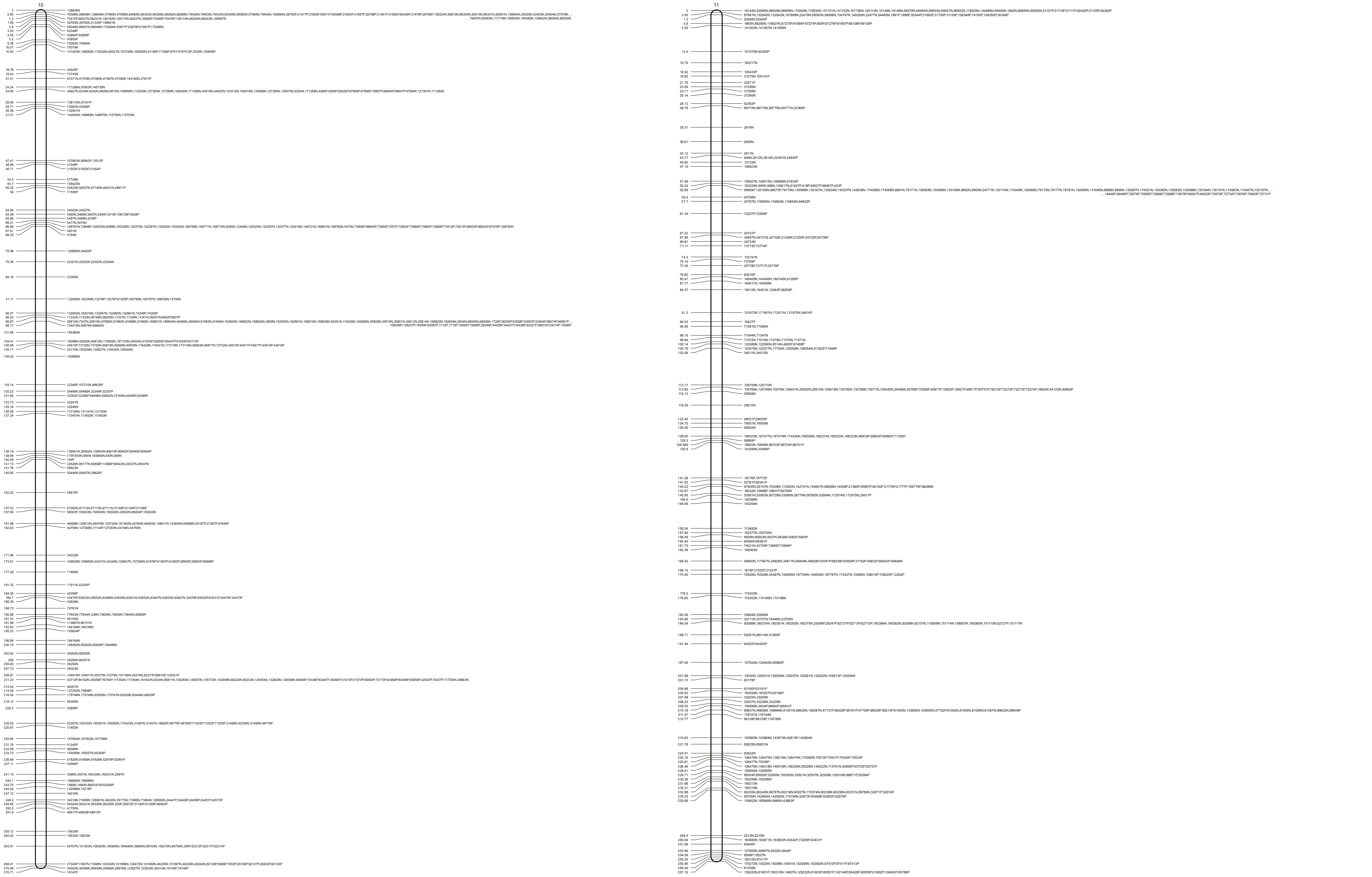
10
11

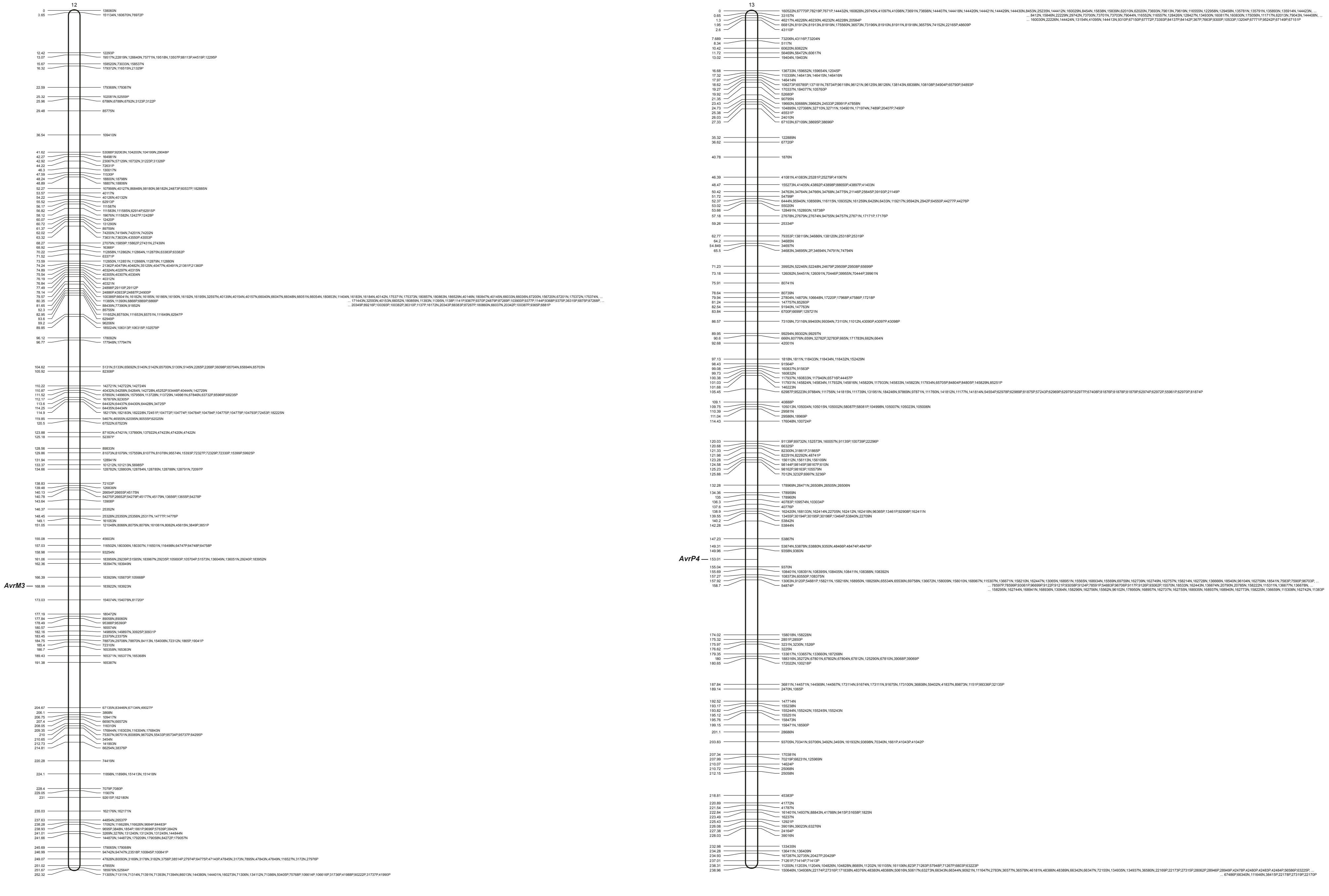

12
13
AvrM3
AvrP4

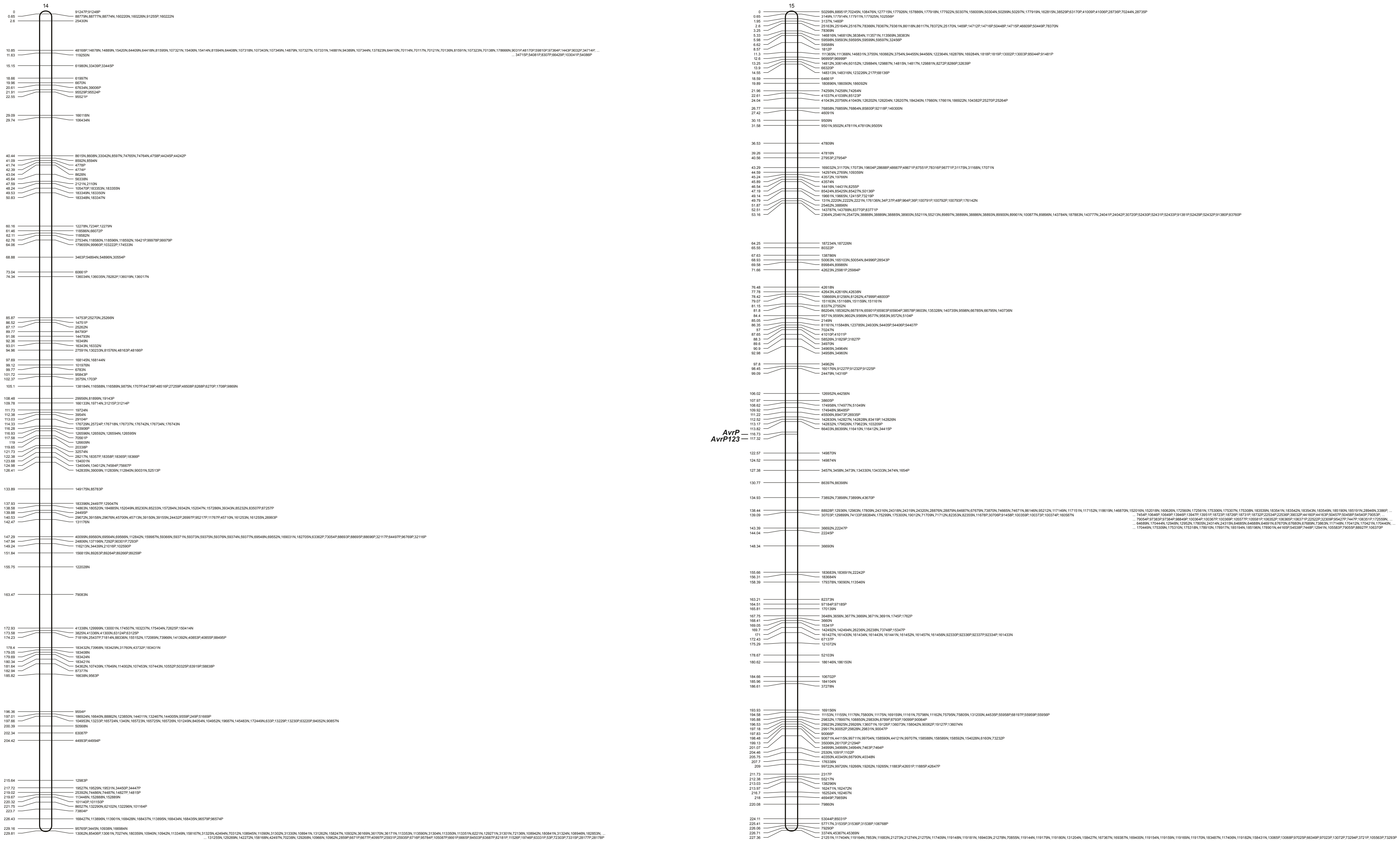
14
15
AvrP
AvrP123

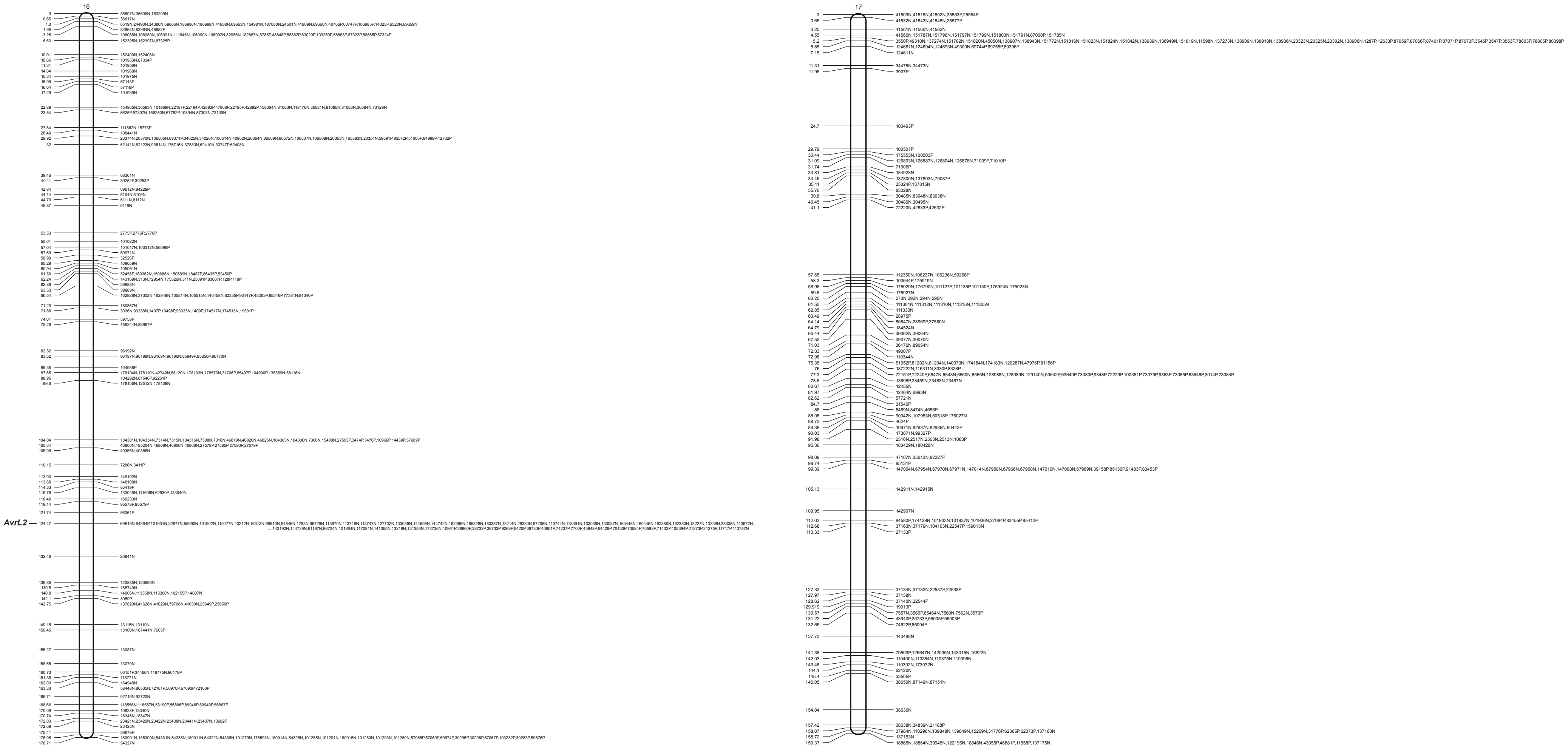
16
AvrL2
17

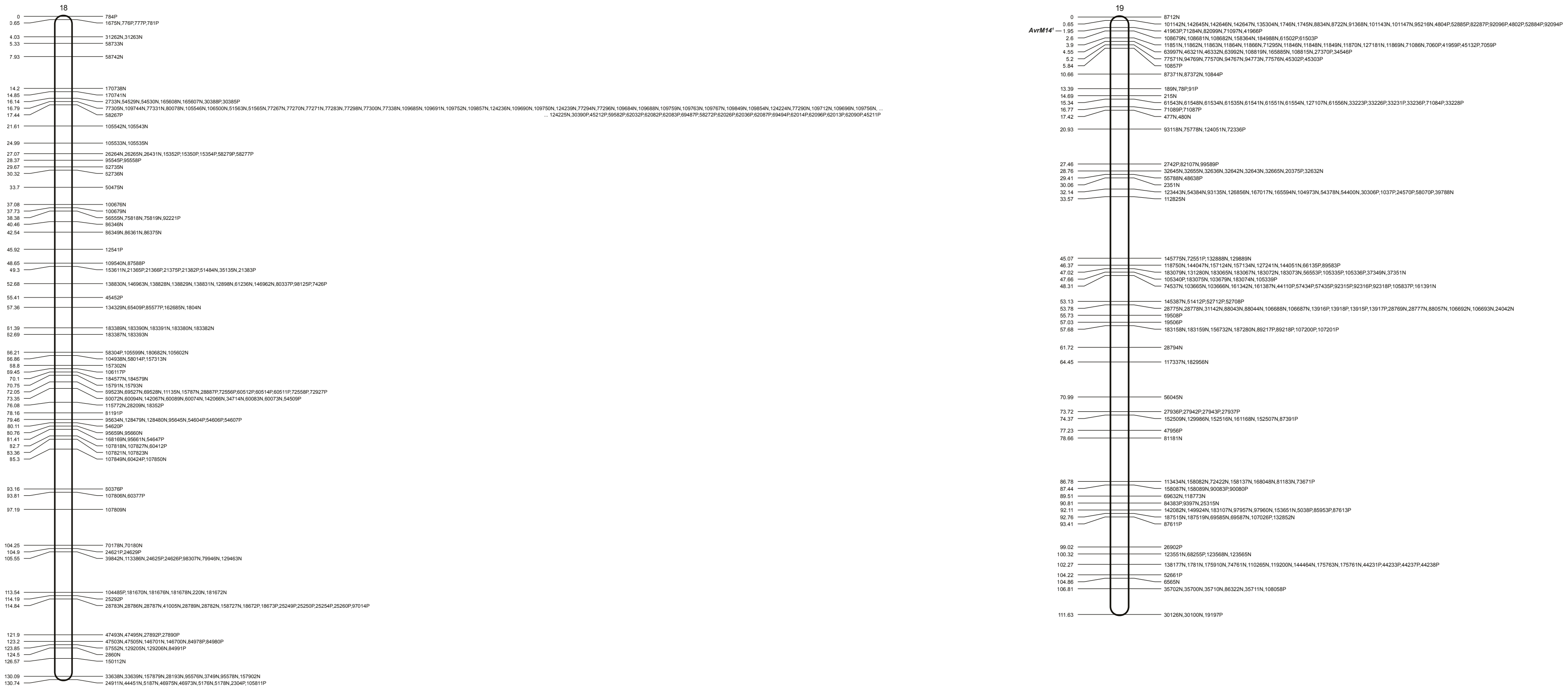
18
19
AvrM14¹

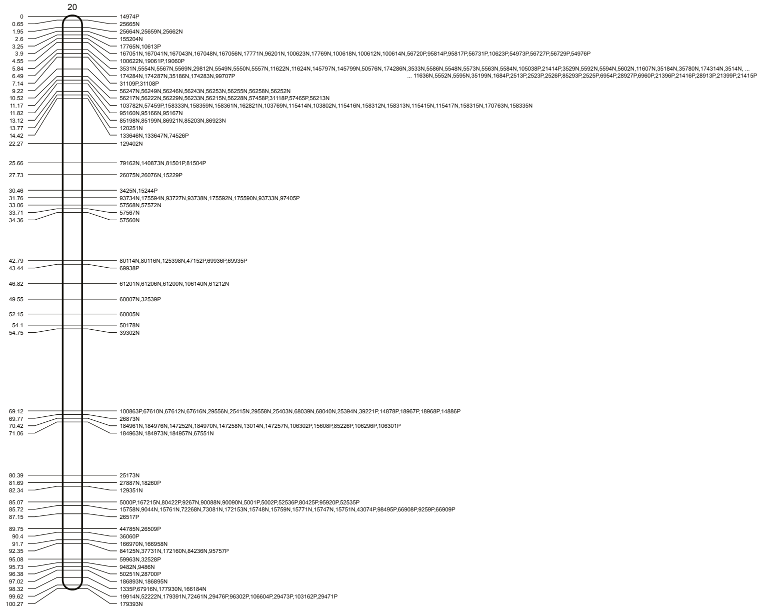
20

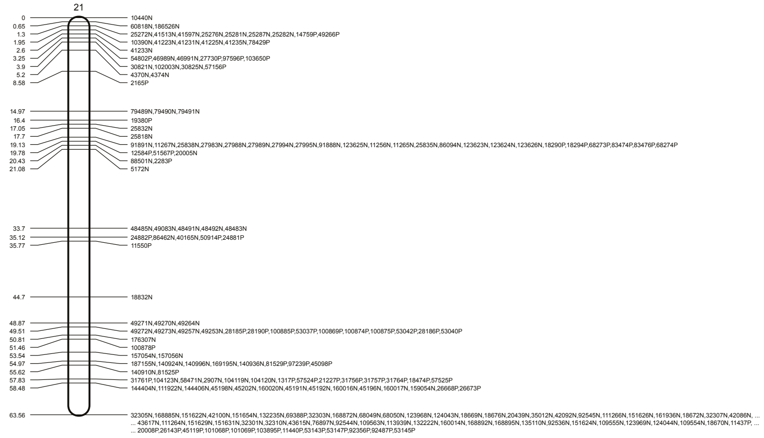
21

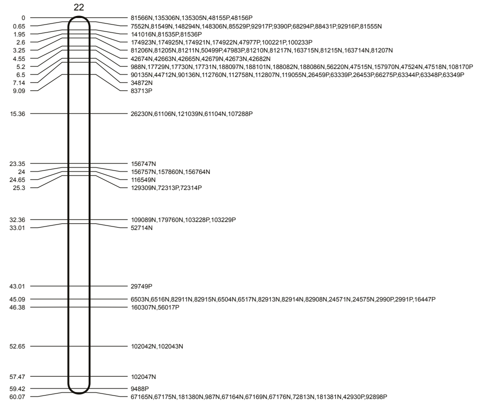
22

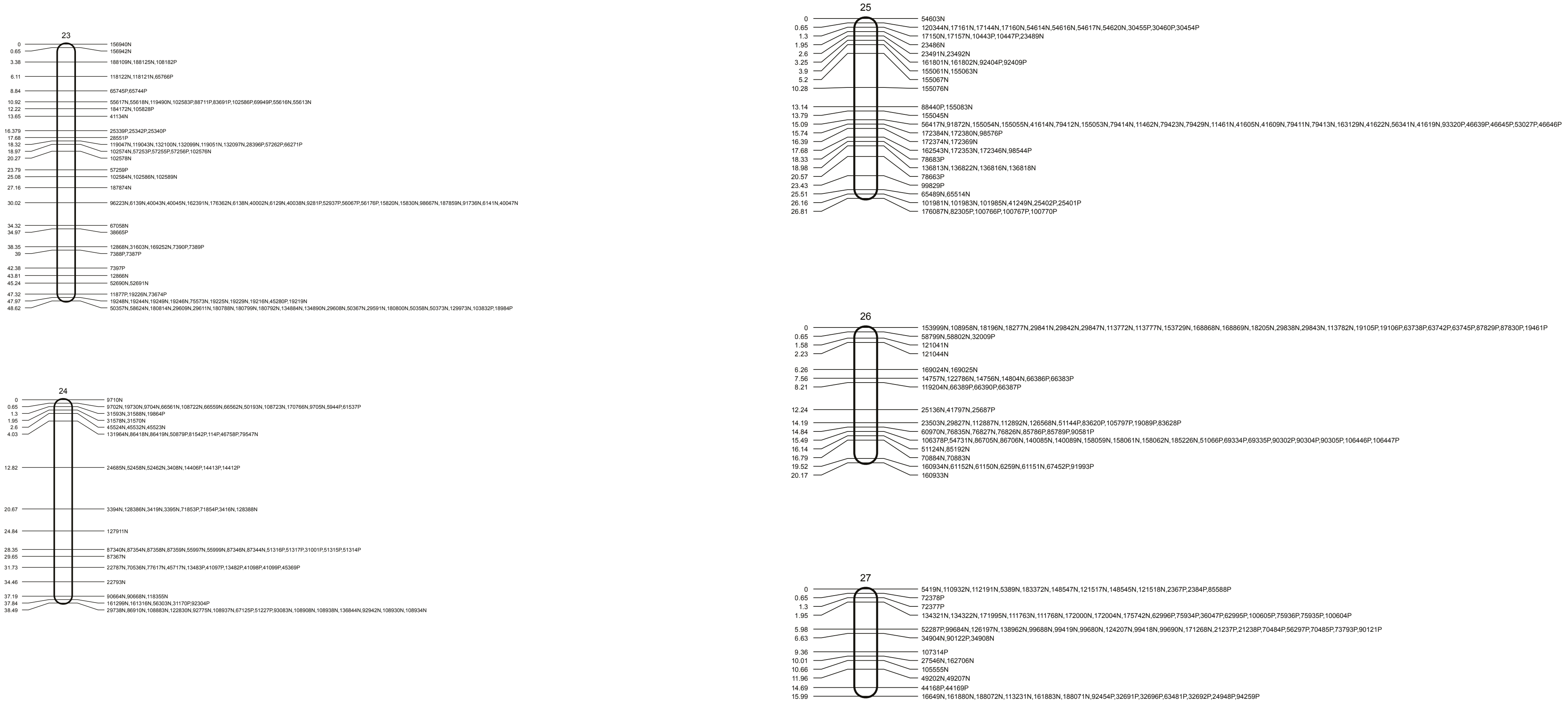
23
0 156940N
0.65 156942N
3.38 188109N,188125N,108182P
6.11 118122N,118121N,65766P
8.84 65745P,65744P
10.92 55617N,55618N,119490N,102583P,88711P,83691P,102586P,69949P,55616N,55613N
12.22 184172N,105828P
13.65 41134N
16.379 25339P,25342P,25340P
17.68 28551P
18.32 119047N,119043N,132100N,132099N,119051N,132097N,28396P,57262P,66271P
18.97 102574N,57253P,57255P,57256P,102576N
20.27 102578N
23.79 57259P
25.08 102584N,102586N,102589N
27.16 187874N
30.02 96223N,6139N,40043N,40045N,162391N,176362N,6138N,40002N,6129N,40038N,9281P,52937P,56067P,56176P,15820N,15830N,98667N,187859N,91736N,6141N,40047N
34.32 67058N
34.97 38665P
38.35 12868N,31603N,169252N,7390P,7389P
39 7388P,7387P
42.38 7397P
43.81 12866N
45.24 52690N,52691N
47.32 11877P,19226N,73674P
47.97 19248N,19244N,19249N,19246N,75573N,19225N,19229N,19216N,45280P,19219N
48.62 50357N,58624N,180814N,29609N,29611N,180788N,180799N,180792N,134884N,134890N,29608N,50367N,29591N,180800N,50358N,50373N,129973N,103832P,18984P
24
0 9710N
0.65 9702N,19730N,9704N,66561N,108722N,66559N,66562N,50193N,108723N,170766N,9705N,5944P,61537P
1.3 31593N,31588N,19864P
1.95 31578N,31570N
2.6 45524N,45532N,45523N
4.03 131964N,86418N,86419N,50879P,81542P,114P,46758P,79547N
12.82 24685N,52458N,52462N,3408N,14406P,14413P,14412P
20.67 3394N,128386N,3419N,3395N,71853P,71854P,3416N,128388N
24.84 127911N
28.35 87340N,87354N,87358N,87359N,55997N,55999N,87346N,87344N,51316P,51317P,31001P,51315P,51314P
29.65 87367N
31.73 22787N,70536N,77617N,45717N,13483P,41097P,13482P,41098P,41099P,45369P
34.46 22793N
37.19 90664N,90668N,118355N
37.84 161299N,161316N,56303N,31170P,92304P
38.49 29738N,86910N,108883N,122830N,92775N,108937N,67125P,51227P,93083N,108908N,108938N,136844N,92942N,108930N,108934N
25
0 54603N
0.65 120344N,17161N,17144N,17160N,54614N,54616N,54617N,54620N,30455P,30460P,30454P
1.3 17150N,17157N,10443P,10447P,23489N
1.95 23486N
2.6 23491N,23492N
3.25 161801N,161802N,92404P,92409P
3.9 155061N,155063N
5.2 155067N
10.28 155076N
13.14 88440P,155083N
13.79 155045N
15.09 56417N,91872N,155054N,155055N,41614N,79412N,155053N,79414N,11462N,79423N,79429N,11461N,41605N,41609N,79411N,79413N,163129N,41622N,56341N,41619N,93320P,46639P,46645P,53027P,46646P
15.74 172384N,172380N,98576P
16.39 172374N,172369N
17.68 162543N,172353N,172346N,98544P
18.33 78683P
18.98 136813N,136822N,136816N,136818N
20.57 78663P
23.43 99829P
25.51 65489N,65514N
26.16 101981N,101983N,101985N,41249N,25402P,25401P
26.81 176087N,82305P,100766P,100767P,100770P
26
0 153999N,108958N,18196N,18277N,29841N,29842N,29847N,113772N,113777N,153729N,168868N,168869N,18205N,29838N,29843N,113782N,19105P,19106P,63738P,63742P,63745P,87829P,87830P,19461P
0.65 58799N,58802N,32009P
1.58 121041N
2.23 121044N
6.26 169024N,169025N
7.56 14757N,122786N,14756N,14804N,66386P,66383P
8.21 119204N,66389P,66390P,66387P
12.24 25136N,41797N,25687P
14.19 23503N,29827N,112887N,112892N,126568N,51144P,83620P,105797P,19089P,83628P
14.84 60970N,76835N,76827N,76826N,85786P,85789P,90581P
15.49 106378P,54731N,86705N,86706N,140085N,140089N,158059N,158061N,158062N,185226N,51066P,69334P,69335P,90302P,90304P,90305P,106446P,106447P
16.14 51124N,85192N
16.79 70884N,70883N
19.52 160934N,61152N,61150N,6259N,61151N,67452P,91993P
20.17 160933N
27
0 5419N,110932N,112191N,5389N,183372N,148547N,121517N,148545N,121518N,2367P,2384P,85588P
0.65 72378P
1.3 72377P
1.95 134321N,134322N,171995N,111763N,111768N,172000N,172004N,175742N,62996P,75934P,36047P,62995P,100605P,75936P,75935P,100604P
5.98 52287P,99684N,126197N,138962N,99688N,99419N,99680N,124207N,99418N,99690N,171268N,21237P,21238P,70484P,56297P,70485P,73793P,90121P
6.63 34904N,90122P,34908N
9.36 107314P
10.01 27546N,162706N
10.66 105555N
11.96 49202N,49207N
14.69 44168P,44169P
15.99 16649N,161880N,188072N,113231N,161883N,188071N,92454P,32691P,32696P,63481P,32692P,24948P,94259P
