# Supplementary material for: Genome analysis and avirulence gene cloning using a high-density RADseq linkage map of the flax rust fungus, Melampsora lini
Source: BMC Genomics. 2016 Aug 22;17(1):667. doi: 10.1186/s12864-016-3011-9 (PMC4994203; doi:10.1186/s12864-016-3011-9)
Supplement: Additional file 5: — Gene segregation ratios in the CH5 F2 mapping family. Segregation ratios of avirulence genes and the I-1 inhibitor gene in the CH5 F2 family as obtained by Lawrence et al. [48]. (DOCX 17 kb) [file 12864_2016_3011_MOESM5_ESM.docx]

| **Avirulence on flax *R* gene** | **Cloned *Avr* gene** | **Reference** | **Segregation ratio (Avr:Vir)^1^ [1]** |
| --- | --- | --- | --- |
| *L1* |  |  | 6:74^2^ |
| *L2* | *AvrL2* | This study | 55:25 |
| *L5* | *AvrL567* | [2] | 59:21 |
| *L6* | *AvrL567* | [2] | 59:21 |
| *L7* | *AvrL567* | [2] | 22:58 |
| *L11* |  |  | 65:15 |
| *M* | *AvrM* | [3] | 63:17 |
| *M1* | *AvrM14* | This study | 18:62 |
| *M3* |  |  | 62:18 |
| *M4* | *AvrM14* | This study | 66:14 |
| *N* |  |  | 60:20 |
| *P* | *AvrP* | [4] | 64:16 |
| *P1* | *AvrP123* | [3,4] | 60:20 |
| *P2* | *AvrP123* | [3,4] | 60:20 |
| *P3* | *AvrP123* | [3,4] | 60:20 |
| *P4* | *AvrP4* | [3] | 64:16 |
| *I-1*^3^ |  |  | 53:24 (inhibitor:non-inhibitor) |

**Additional file 5. Gene segregation ratios in CH5 F_2_ mapping family.**

^1^ The F_2_ family described by Lawrence et al. [1] comprised 80 individuals. Of these, 76 were used in the current study along with one additional F_2_ individual.

^2^ Of the 74 individuals virulent on *L1* flax, only 18 could be confirmed as homozygous for the virulent *avrL1* allele as 52 individuals contained *I-1* and the *I-1* genotype could not be determined in a further four individuals due to lack of *AvrL567* and *AvrM14*.

^3^ *I-1* genotypes were inferred based on the infection phenotypes of F_2_ individuals inoculated onto *L7* and *M1* flax compared to *L5*, *L6* and *M4* flax. Avirulence on *L7* is encoded by the same gene as avirulence on *L5* and *L6* (*AvrL567*; [2]), yet only recognition by *L7* flax is prevented in the presence of the *I-1* avirulence inhibitor. Similarly, avirulence on *M1* and *M4* co-segregate [1] and are encoded by a single gene (*AvrM14*; this paper), yet only avirulence recognition by *M1* flax is prevented in the presence of the *I-1* avirulence inhibitor. Therefore, the *I-1* genotype can be inferred for individuals containing either of these avirulence genes.

**References**

[1] Lawrence GJ, Mayo GME, Shepherd KW. Interactions between genes controlling pathogenicity in the flax rust fungus. Phytopathol. 1981;71:12-19.

[2] Dodds PN, Lawrence GJ, Catanzariti A-M, Ayliffe MA, Ellis JG. The *Melampsora lini AvrL567* avirulence genes are expressed in haustoria and their products are recognized inside plant cells. Plant Cell. 2004;16:755-768.

[3] Catanzariti A-M, Dodds PN, Lawrence GJ, Ayliffe MA, Ellis JG. Haustorially expressed secreted proteins from flax rust are highly enriched for avirulence elicitors. Plant Cell. 2006;18:243-256.

[4] Barrett LG, Thrall PH, Dodds PN, van der Merwe M, Linde CC, Lawrence GJ, Burdon JJ. Diversity and evolution of effector loci in natural populations of the plant pathogen *Melampsora lini*. Mol Biol Evol. 2009;26:2499-2513.
