# Supplementary material for: Genome analysis and avirulence gene cloning using a high-density RADseq linkage map of the flax rust fungus, Melampsora lini
Source: BMC Genomics. 2016 Aug 22;17(1):667. doi: 10.1186/s12864-016-3011-9 (PMC4994203; doi:10.1186/s12864-016-3011-9)
Supplement: Additional file 7: — Within-linkage group chimeric scaffolds. Comparison of physical and genetic maps for scaffolds that are: (A) within-linkage group chimeras; or (B) within- and between-linkage group chimeras. The positions of genetic markers are shown as vertical bars on the physical maps and these are joined to their corresponding positions in the genetic maps by a dotted line. For each gap in the genetic map, the number of markers shown represents those present in recombination bins that lie between the bins containing the markers that flank the chimeric breakpoint. (PDF 838 kb) [file 12864_2016_3011_MOESM7_ESM.pdf]

A

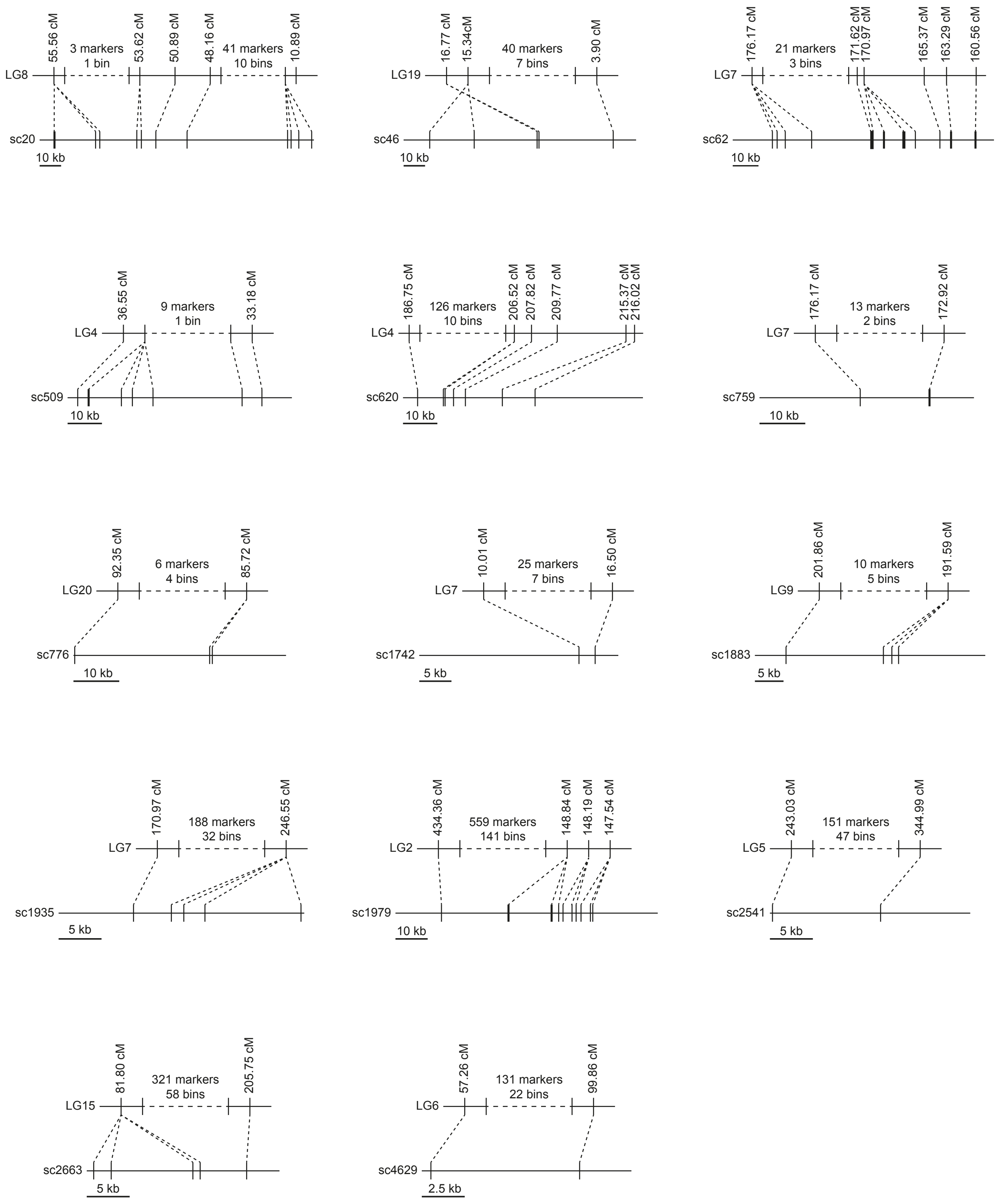

B

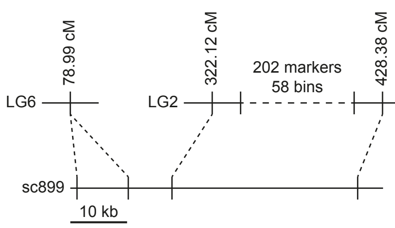

Additional file 7. Within-linkage group chimeric scaffolds. Comparison of physical and genetic maps for scaffolds that are: (A) within-linkage group chimeras; or (B) within- and between-linkage group chimeras. The positions of genetic markers are shown as vertical bars on the physical maps and these are joined to their corresponding positions in the genetic maps by a dotted line. For each gap in the genetic map, the number of markers shown represents those present in recombination bins that lie between the bins containing the markers that flank the chimeric breakpoint.
