# Supplementary material for: Genome analysis and avirulence gene cloning using a high-density RADseq linkage map of the flax rust fungus, Melampsora lini
Source: BMC Genomics. 2016 Aug 22;17(1):667. doi: 10.1186/s12864-016-3011-9 (PMC4994203; doi:10.1186/s12864-016-3011-9)
Supplement: Additional file 9: — Gene, repeat and nucleotide content in regions of the M. lini genome with different recombination rates. The 2756 recombination bins in the CH5 genetic map were ordered by increasing cumulative scaffold length and grouped to produce ten genome fractions of approximately equal physical size (mean 11,654 kb), with group 1 representing regions of the genome with the highest recombination rate and group 10 representing regions of the genome with the lowest recombination rate. Gene density, effector density and the percentage of GC, N and repeat-masked DNA were then determined for each genome fraction. (DOCX 15 kb) [file 12864_2016_3011_MOESM9_ESM.docx]

| Genome fraction | Cumulative scaffold length per bin (kb) | Number of bins | Total sequence (kb) | Genes per 100 kb | Effectors per Mb | % GC | % N | % Repetitive DNA |
| --- | --- | --- | --- | --- | --- | --- | --- | --- |
| 1 | 0 - 19.35 | 1,184 | 11,656 | 9.92 | 5.78 | 40.34 | 10.81 | 19.47 |
| 2 | 19.36 - 32.31 | 462 | 11,644 | 9.47 | 5.73 | 40.65 | 11.25 | 20.34 |
| 3 | 32.32 - 45.00 | 305 | 11,667 | 9.58 | 7.10 | 41.02 | 11.39 | 21.90 |
| 4 | 45.10 - 60.43 | 223 | 11,641 | 9.17 | 6.60 | 41.05 | 12.15 | 22.76 |
| 5 | 60.51 - 76.00 | 173 | 11,688 | 8.30 | 5.66 | 40.96 | 11.73 | 23.88 |
| 6 | 76.24 - 93.66 | 138 | 11,655 | 8.99 | 4.95 | 41.16 | 12.46 | 23.72 |
| 7 | 93.82 - 121.78 | 110 | 11,695 | 8.43 | 4.50 | 41.04 | 13.03 | 25.53 |
| 8 | 121.79 - 170.49 | 82 | 11,585 | 8.09 | 4.23 | 41.24 | 12.32 | 26.16 |
| 9 | 173.50 - 314.50 | 54 | 11,621 | 7.83 | 4.75 | 41.23 | 12.99 | 27.86 |
| 10 | 318.60 - 754.19 | 25 | 11,692 | 8.29 | 3.06 | 41.04 | 10.51 | 26.49 |

**Additional file 9. Gene, repeat and nucleotide content in regions of the *M. lini* genome with different recombination rates.**

The total nucleotide content, repeat content and gene/effector content of scaffolds associated with each recombination bin was determined as described in Additional file 2 and as shown in Additional file 8. The 2,756 recombination bins were ordered by increasing cumulative scaffold length and grouped to produce ten genome fractions of approximately equal physical size (mean 11,654 kb), with group 1 representing regions of the genome with the highest recombination rate and group 10 representing regions of the genome with the lowest recombination rate. Gene density, effector density and the percentage of GC, N and repeat-masked DNA were then determined for each genome fraction.
