# Supplementary material for: Genome analysis and avirulence gene cloning using a high-density RADseq linkage map of the flax rust fungus, Melampsora lini
Source: BMC Genomics. 2016 Aug 22;17(1):667. doi: 10.1186/s12864-016-3011-9 (PMC4994203; doi:10.1186/s12864-016-3011-9)
Supplement: Additional file 10: — Loss of heterozygosity in MS48. Graphs showing: (A) read depth and percentage of H allele reads per marker across the CH5 genetic map; and (B) mean read depth and mean percentage of H allele reads per marker in regions of chromosome duplication or deletion in the loss-of-AvrM14 mutant, MS48. (PDF 139 kb) [file 12864_2016_3011_MOESM10_ESM.pdf]

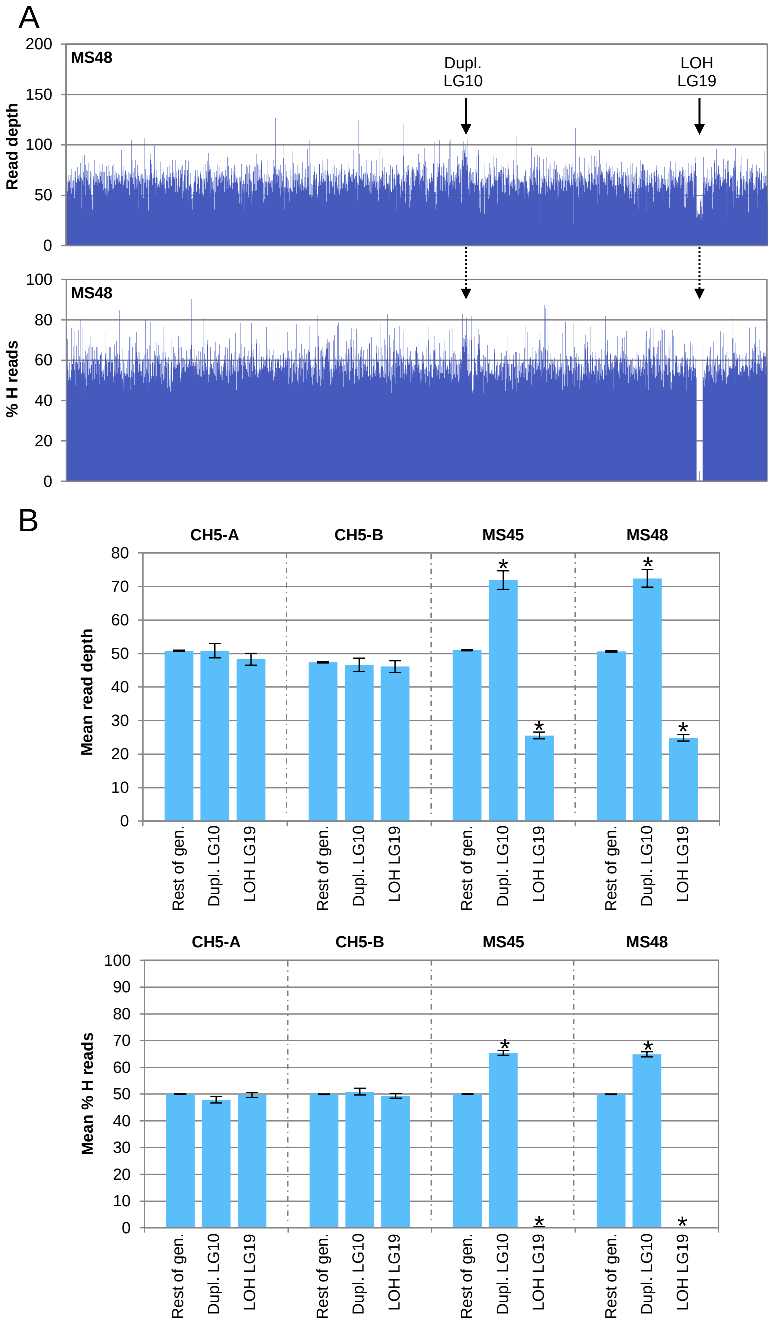

Additional file 10. Loss of heterozygosity in MS48.

(A) Read depth and percentage of H allele reads per marker across the CH5 genetic map. Markers are ordered according to their position in the linkage group. Linkage groups are shown in numerical order, which was assigned according to linkage group size and may not reflect relative position in the genome. Identified regions showing chromosome duplication (Dupl.) or loss of heterozygosity (LOH) are marked by black arrows. Identified regions in MS48 are as follows: Dupl. LG10 – 64 *Nsi*I markers located between 0 and 10.93 cM; LOH LG19 – 80 *Nsi*I markers located between 0 and 32.14 cM. (B) Mean read depth and mean percentage of H allele reads per marker in identified regions compared to the rest of the genome. Significant differences between identified regions and the rest of the genome for a given individual are marked with an asterisk (two-tailed T-test; p <0.001). Error bars represent ± one standard error. CH5-A and CH5-B are duplicate CH5 samples.
