# Supplementary material for: Genome analysis and avirulence gene cloning using a high-density RADseq linkage map of the flax rust fungus, Melampsora lini
Source: BMC Genomics. 2016 Aug 22;17(1):667. doi: 10.1186/s12864-016-3011-9 (PMC4994203; doi:10.1186/s12864-016-3011-9)
Supplement: Additional file 11: — Chromosome duplication in CH5F2-133. Graphs showing: (A) read depth and percentage of H allele reads per marker across the CH5 genetic map; and (B) mean read depth and mean percentage of H allele reads per marker in a region of chromosome duplication in the CH5 F2 individual, CH5F2-133. (PDF 137 kb) [file 12864_2016_3011_MOESM11_ESM.pdf]

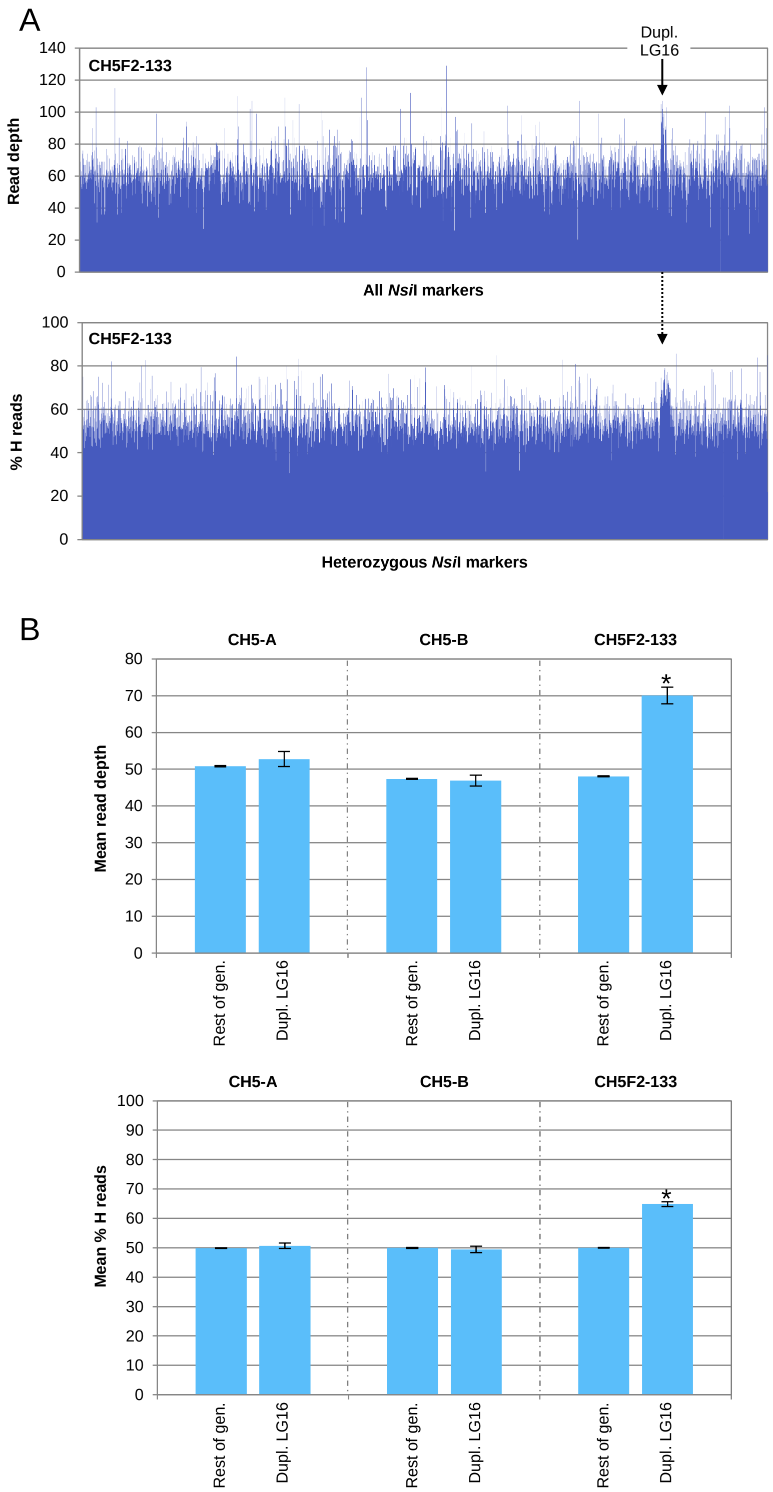

Additional file 11. Chromosome duplication in CH5F2-133.
(A) Read depth and percentage of H allele reads per marker across the CH5 genetic map. Markers are ordered according to their position in the linkage group. Linkage groups are shown in numerical order, which was assigned according to linkage group size and may not reflect relative position in the genome. The region showing chromosome duplication (Dupl.) is marked and corresponds to 72 consecutive *Nsi*I markers in LG16 located between 113.68 and 158.65 cM. (B) Mean read depth and mean percentage of H allele reads per marker in the duplicated region of CH5F2-133 compared to the rest of the genome. Significant differences between the identified region and the rest of the genome for a given individual are marked with an asterisk (two-tailed T-test; $p < 0.001$). Error bars represent ± one standard error. CH5-A and CH5-B are duplicate CH5 samples. Note that for (A) and (B) the read depth information shown corresponds to all *Nsi*I markers scored in these three individuals, whereas the percentage of H allele reads corresponds to only those markers that were scored as heterozygous in CH5F2-133.
