# Supplementary material for: Genome analysis and avirulence gene cloning using a high-density RADseq linkage map of the flax rust fungus, Melampsora lini
Source: BMC Genomics. 2016 Aug 22;17(1):667. doi: 10.1186/s12864-016-3011-9 (PMC4994203; doi:10.1186/s12864-016-3011-9)
Supplement: Additional file 12: — AvrM14 sequences. DNA sequences of AvrM14-A and AvrM14-B and their alignment to sc27. (PDF 377 kb) [file 12864_2016_3011_MOESM12_ESM.pdf]

```
                         C34-1
sc27          CACATTGATCCCGTTCATAACATATTTCTACATAACCTTCCCTCACGATATTCGCTCTTT   1582
AvrM14-A      ---------------------ATATTTCTACATAACCTTCCCTCACGATATTCGCTCTTT   39
AvrM14-B      ---------------------ATATTTCTACATAACCTTCCCTCACGATATTCGCTCTTT   39

sc27          CAAAAATGAAGTTTGGTATCATCTTTTTAGCCCTCTTCTTTGTCACTATCAGTCACGTTC   1642
AvrM14-A      CAAAAATGAAGTTTGGTATCATCTTTTTAGCCCTCTTCTTTGTCACTATCAGTCACGTTC   99
AvrM14-B      CAAAAATGAAGTTTGGTATCATCTTTTTAGCCCTCTTCTTTGTCACTATCAGTCACGTTC   99

sc27          TATGCGCCGGCAATAATGATCTTGAAAATGTGAAGGCAATCGCGATAGTTTACAGGGAGC   1702
AvrM14-A      TATGCGCCGGCAATAATGATCTTAAAAATGTGAAGGCAATCGCGATAGTTTACAGGGAGC   159
AvrM14-B      TATGCGCCGGCAATAATGATCTTGAAAATGTGAAGGCAATCGCGATAGTTTACAGGGAGC   159

sc27          TCTCAGACGGGTCTCAGACAGTCCTCCTGGCGAAAATGAAAGGCCTGGCGGACGAGAAAG   1762
AvrM14-A      TCTCAGACGGGTCTCAGACAGTCCTCCTGGCGAAAATGAAAGGCCTGGCGGACGAGAAAG   219
AvrM14-B      TCTCAGACGGGTCTCAGACAGTCCTCCTGGCGAAAATGAAAGGCCTGGCGGACGAGAAAG   219

                                        BspEI
sc27          GATGGATGTTTGTAAGAGGACATGTCCGGAAGGACGAGGAGGCTGACCCTGGGGTCGCCG   1822
AvrM14-A      GATGGATGTTTGTAAGAGGACATGTCCGGGAGGACGAGGAGGCTGACCCTGGGGTCGCCG   279
AvrM14-B      GATGGATGTTTGTAAGAGGACATGTCCGGAAGGACGAGGAGGCTGACCCTGGGGTCGCCG   279

sc27          CGATACGCGAAACGCAGGAAGAAAGTCAGTTAACAGACATTCCAGCCTTTCAGAGAGCAA   1882
AvrM14-A      CGATACGCGAAACGCAGGAAGAAAGTCAGTTAACAGACATTCCAGCCTTTCAGAGAGCAA   339
AvrM14-B      CGATACGCGAAACGCAGGAAGAAAGTCAGTTAACAGACATTCCAGCCTTTCAGAGAGCAA   339

sc27          ACTTTGGAGCTTTACTAACCCTCAGTCCTTCTCGACATCAGCCGGCTTTACAGGAATGGT   1942
AvrM14-A      ACTTTGGAGCTTTACTAACCCTCAGTCCTTCTCGACATCAGCCGGCTTTACAGGAATGGT   399
AvrM14-B      ACTTTGGAGCTTTACTAACCCTCAGTCCTTCTCGACATCAGCCGGCTTTACAGGAATGGT   399

sc27          GAAGAAAAGCGGCGCACCGTTCACCCAACCGGGTTCCAAAGATACTCAGCGTGTTATCAC   2002
AvrM14-A      GAAGAAAAGCGGCGCACCGTTCACCCAACCGGGTTCCAAAGATACTCAGCGTGTTATCAC   459
AvrM14-B      GAAGCAAAGCGGCGCACCGTTCACCCAACCGGGTTCCAAAGATCCTCAGCGTGTTATCAC   459

sc27          AATACACCCTCATATTGTCCAAGTTCAAGAGGCTTCAAAGTCTAAGGACACAGAAGACAC   2062
AvrM14-A      AATACACCCTCATATTGTCCAAGTTCAAGAGGCTTCA-----------------------   496
AvrM14-B      AATACACCCTCATATTGTCCAAGTTCAAGAGGCTTCA-----------------------   496

sc27          AGTCAAACGTCAATTTCTCTGGGTCCNNNNNNNNNNNNNNNNNNNNNNNNNNNNNNAAAG   2122
AvrM14-A      ------------------------------------------------------------   496
AvrM14-B      ------------------------------------------------------------   462

sc27          CGGCGCACCGTTCACCCAACCGGGTTCCAAAGATCCTCAGCGTGTTATCACAATACACCC   2182
AvrM14-A      ------------------------------------------------------------   496
AvrM14-B      ------------------------------------------------------------   496

sc27          TCATATTGTCCAAGTTCAAGAGGCTTCAAAGTCTAAGGACACAGAAGACACAGTCAAACG   2242
AvrM14-A      ----------------------------AAGTCTAAGGACACAGAAGACACAGTCAAACG   528
AvrM14-B      ----------------------------AAGTCTAAGGACACAGAAGACACAGTCAAACG   528

sc27          CGAATTTCTCTGGGTCCGCCCATCCGAGGTCCGAAGTAAGCTACAAAGGGCGGAAATGAT   2302
AvrM14-A      TCAATTTCTCTGGGTCCACCCATCCGAGGTCCGAAGTAAGCTACAAAGGGCGGAAATGAT   588
AvrM14-B      CGAATTTCTCTGGGTCCGCCCATCCGAGGTCCGAAGTAAGCTACAAAGGGCGGAAATGAT   588

sc27          TCAAGCCTGGGATCAACTACACAGCTTTTTCTGAGAAACCTCGATGAAGTAAGTGTTGCA   2362
AvrM14-A      TCAAGCCTGGGATCAACTACACAGCTTTTTCTGAGAAACCTCGATGAAGTAAGTGTTGCA   648
AvrM14-B      TCAAGCCTGGGATCAACTACACAGCTTTTTCTGAGAAACCTCGATGAAGTAAGTGTTGCA   648

sc27          AGTGTTCGAATTGCCGCGGATCCATGATTGAATATCAACTGATATAGGGTCGGCTGTAGG   2422
AvrM14-A      AGTGTTCGAATTGCCGCGGATCCATGATTGAATATCAACTGATATAGGGTCGGCTGTAGG   708
AvrM14-B      AGTGTTCGAATTGCCGCGGATCCATGATTGAATATCAACTGATATAGGGTCGGCTGTAGG   708

sc27          ACAACGCTAGGTAGAGAACCGGAACGGTTCTTTGTGGCTCGATCCTTTACCACCTCAGGC   2482
AvrM14-A      ACAACGCTAGGTAGAGAACCGGAACGGTTCTTTGTGGCTCGATCCTTTACCACCTCAGGC   768
AvrM14-B      ACAACGCTAGGTAGAGAACCGGAACGGTTCTTTGTGGCTCGATCCTTTACCACCTCAGGC   768

sc27          TACAGTATAAAACCTTTTATCAAAAGACGACTACTATTCAATCATTTAATTCATTCCTTT   2542
AvrM14-A      TACAGTATAAAACCTTTTATCAAAAGACGACT----------------------------   800
AvrM14-B      TACAGTATAAAACCTTTTATCAAAAGACGACT----------------------------   800
                  C34-4
sc27          GTTCCGAAAATC 2554
AvrM14-A      ------------ 800
AvrM14-B      ------------ 800
```

Additional file 12. ***AvrM14*** **sequences.**
Alignment of *AvrM14-A* and *AvrM14-B* with the corresponding region of sc27. Note that the scaffold sequence contains 171 bp of additional sequence that is not present in the *AvrM14-A* and *AvrM14-B* genes. It is likely that this additional sequence prevented gene prediction by the annotation pipeline used by Nemri et al. [1]. As a result, *AvrM14* is not present in the list of *M. lini* effectors predicted by these authors. The C34-1 and C34-4 primer sites are shaded green, polymorphic nucleotides are shaded blue and a 77 bp intron is shaded yellow. Start (ATG) and stop (TGA) codons are shown in bold type. The position of a polymorphic *Bsp*EI restriction site is also shown.

References

[1] Nemri A, Saunders DGO, Anderson C, Upadhyaya NM, Win J, Lawrence GJ, et al. The genome sequence and effector complement of the flax rust pathogen *Melampsora lini*. Front Plant Sci. 2014;5:98.
