# Supplementary material for: Genome analysis and avirulence gene cloning using a high-density RADseq linkage map of the flax rust fungus, Melampsora lini
Source: BMC Genomics. 2016 Aug 22;17(1):667. doi: 10.1186/s12864-016-3011-9 (PMC4994203; doi:10.1186/s12864-016-3011-9)
Supplement: Additional file 13: — Agroinfiltration of avirulence gene constructs. The response of flax cultivars and near-isogenic lines to expression of avirulence gene candidates (AvrM14-A, AvrM14-B and AvrL2-A) using Agrobacterium tumefaciens-mediated transient transformation. (PDF 2637 kb) [file 12864_2016_3011_MOESM13_ESM.pdf]

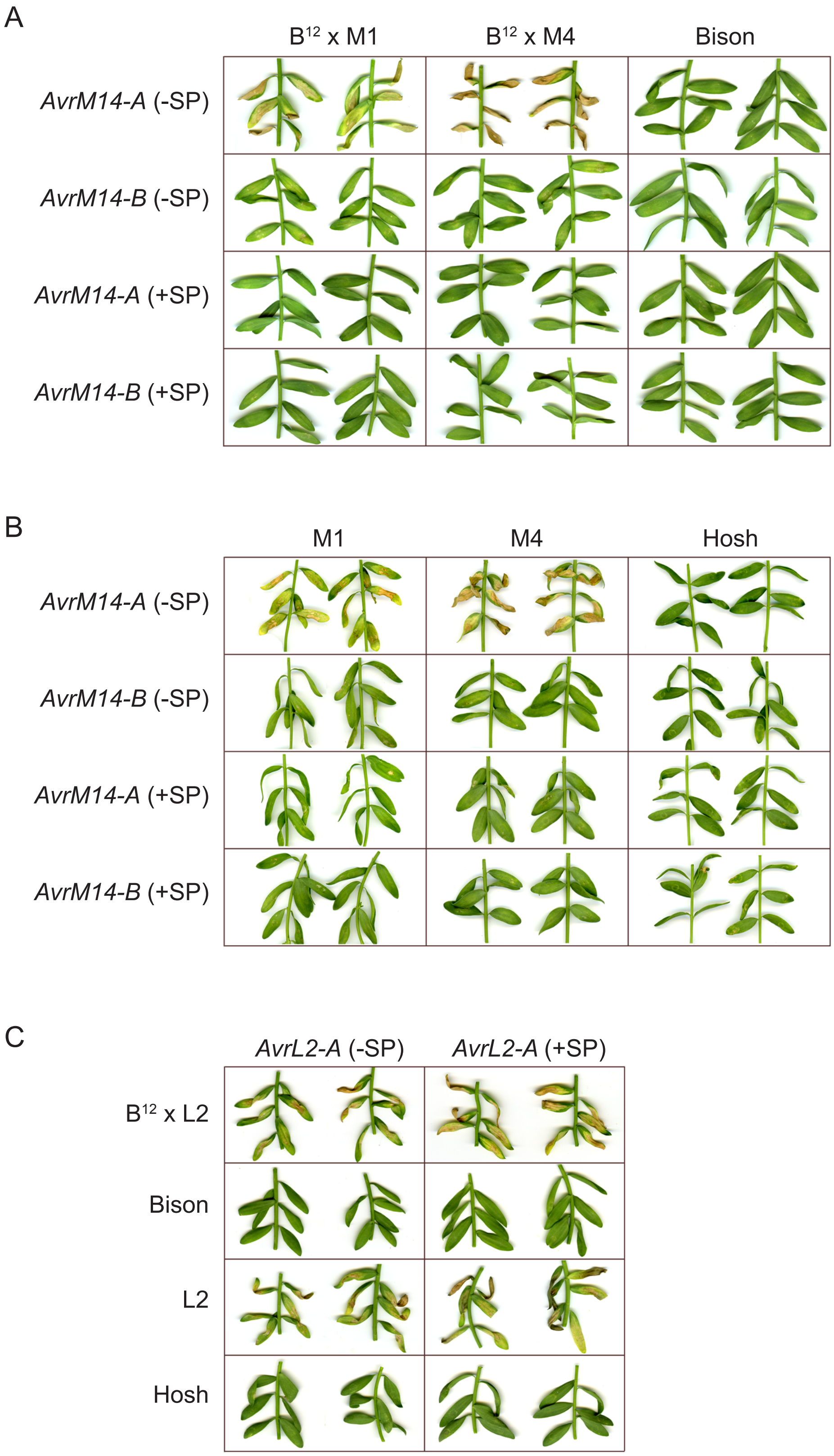

Additional file 13. Agroinfiltration of avirulence gene constructs.

Contructs containing (+SP) or lacking (-SP) the region encoding the signal peptide were transiently expressed in flax leaves using *Agrobacterium tumefaciens*-mediated transient transformation. Photographs were taken 8 days after infiltration. (A) and (B) Avirulence allele derived from strain H (*AvrM14-A*) and virulence allele derived from strain C (*AvrM14-B*) expressed (A) in near-isogenic lines containing *M1* (B^{12} x M1), *M4* (B^{12} x M4) or lacking both resistance genes (Bison) or (B) in cultivars containing *M1* (Williston Brown; shown as M1), *M4* (Victory A; shown as M4) or lacking both resistance genes (Hoshangabad; shown as Hosh). (C) Avirulence allele derived from strain H (*AvrL2-A*) expressed in near-isogenic lines containing (B^{12} x L2) or lacking *L2* (Bison), or in cultivars containing (Stewart; shown as L2) or lacking *L2* (Hoshangabad; shown as Hosh). The -SP version of *AvrL2-A* lacked the region encoding the first 36 amino acids.
