# Supplementary material for: Genome analysis and avirulence gene cloning using a high-density RADseq linkage map of the flax rust fungus, Melampsora lini
Source: BMC Genomics. 2016 Aug 22;17(1):667. doi: 10.1186/s12864-016-3011-9 (PMC4994203; doi:10.1186/s12864-016-3011-9)
Supplement: Additional file 14: — RADseq markers that co-segregate with AvrL2 and for which only the strain C allele is present in CH5F2-105, MS32 and MS38. Table showing marker sequences and their scaffold location in the M. lini genome assembly. (DOCX 16 kb) [file 12864_2016_3011_MOESM14_ESM.docx]

| **Marker name** | **Discovery method** | **Marker type^1^** | **Identity** | **Scaffold** | **Scaffold coordinates** | **Orientation** | **Scaffold length** | **Consensus RADtag sequence (5’-3’)** |
| --- | --- | --- | --- | --- | --- | --- | --- | --- |
| 1866N-SE | de novo assembly | ab/aa | 92/92 bp | sc4334 | 10,858-10,949 | - | 13,347 | TGCATTTGCTCCACGGCGGTCATGCGGTAGATATCATTTTGATTCAAGTACAATTGTAATCACCTACCCTCATCAGATGATGCAGTTCTCAA |
| 14067P-SE^2^ | de novo assembly | ab/aa | 91/92 bp | sc4334 | 5,684-5,775 | + | 13,347 | TGCAGGTGTAGAGATGAGACATGAGTCTGTTAAAGATGTTTGGTTGATACCTTTCTAGTGGTGAGCATGACTGCAAGATCAAGATGTTTGAT |
| 65889P^2^ | reference-aligned | ab/aa | 91/92 bp | sc4334 | 5,684-5,775 | + | 13,347 | TGCAGGTGTAGAGATGAGACATGAGTCTGTTAAAGATGTTTGGTTGATACCTTTCTAGTGGTGAGCATGACTGCAAGATCAAGATGTTTGAT |
| 12773N-SE | de novo assembly | ab/ac | 88/92 bp | Multiple^3^ | n/a | n/a | n/a | TGCATACCAGTAGTGGAAGAAAGAATTCAACACATCAAAGAAGTGCAAAAGGAATTGAAAGATGCGATTAACCTATCACAAGAAGAAATGAA |
| 13632P-SE | de novo assembly | ab/ac | 85/90 bp | Multiple^4^ | n/a | n/a | n/a | TGCAGTGTTCATTGAATGTACTATAATGTCATCAATGTATACTGCCATCCATCCTTCTCTTATTTCTTTACAAAATGTTCTATCCATGGTTC |

**Additional file 14. RADseq markers that co-segregate with *AvrL2* and for which only the strain C allele is present in CH5F2-105, MS32 and MS38.**

^1^ Marker types are shown as xx/yy where xx represents the genotype of strain H and yy represents the genotype of strain C

^2^ Markers 14067P_SE and 65889P are identical. These markers were heterozygous in all avirulent F_2_ individuals suggesting that they detect a polymorphism between adjacent copies of a homologous sequence rather than between alleles, yet only one copy was present in the CH5 assembly

^3^ Marker 12773N_SE matched 14 different scaffolds with 88/92 bp identity

^4^ Marker 13632P_SE matched five different scaffolds with 85/90 bp identity
