# Supplementary material for: Genome analysis and avirulence gene cloning using a high-density RADseq linkage map of the flax rust fungus, Melampsora lini
Source: BMC Genomics. 2016 Aug 22;17(1):667. doi: 10.1186/s12864-016-3011-9 (PMC4994203; doi:10.1186/s12864-016-3011-9)
Supplement: Additional file 15: — AvrL2 family DNA sequences. Alignment of AvrL2 coding regions with their closest matching scaffolds in the M. lini genome assembly. Polymorphic nucleotides are shaded. (PDF 337 kb) [file 12864_2016_3011_MOESM15_ESM.pdf]

```
AvrL2-A     ATGGGCAAAGGAAATAACATTCAAACGCCGTGCTTTCGTGCATCTCAACTTCGAAGCTTT 60
sc8713_1    ------------------------------------------------------------ 0
sc8713_2    ATGGGCAAAGGAAATAACATTCAAACGCCGTGCTTTCGTGCATCTCAACTTCGAAGCTTT 3146

AvrL2-A     TGTTTAATTGCTTTCTTATTGTGTCAATCCCTTCAATCCATCGTCTCACTGCCAGCACTT 120
sc8713_1    ------------------------------------------------------------ 0
sc8713_2    TGTTTAATTGCTTTCTTATTGTGTCAATCCCTTCAATCCATCGTCTCACTGCCAGCACTT 3206

AvrL2-A     TCCTCCAAAGTGGAGTTGTCAGCACAAAAAATTAAGGTACAAGCACGGGTGAATCAGTTT 180
sc8713_1    ---------------------------------------------------AATCAGTTT 9
sc8713_2    TCCTCCAAAGTGGAGTTGTCAGCACAAAAAATTAAGGTACAAGCACGGGTGAATCAGTTT 3266

AvrL2-A     GTGAGAGAAAACAACCGTCCCCCTCGTCGAAGTGAACTCGATGTCATCCTTAGCCAGGAA 240
sc8713_1    GTGAGAGAAAACAACCGTCCCCCTCGTCGAAGTGAACTCGATGTCATCCTTAGCCAGGAA 69
sc8713_2    GTGAGAGAAAACAACCGTCCCCCTCG---------------------------------- 3292

AvrL2-A     AATCTCAAACTGAAAGCAGGTAAGCAATAAGACTTATATCACTATCACTATCTGAGCACT 300
sc8713_1    AATCTCAAACTGAAAGCAGGTAAGCAATAAGACTTATATCACTATCACTATCTGAGCACT 129
sc8713_2    ------------------------------------------------------------ 3292

AvrL2-A     GATCCCAATCTTCTTCGACGCGGGTATCCAGGTTATCTTCCCCCGCAATCGTCAACGTCG 360
sc8713_1    GATCCCAATCTTCTTCGACGCGGGTATCCAGGTTATCTTCCCCCGCAATCGTCAACGTCG 189
sc8713_2    ------------------------------------------------------------ 3292

AvrL2-A     CACGAGGACAATTGGGAAACTCTTGACAAGGAAATTGAGGAATATCGCAATGGCAAAAGT 420
sc8713_1    CACGAGGACAATTGGGAAACTCTTGACAAGGAAATTGAGGAATATCGCAATGGCAAAAGT 249
sc8713_2    ------------------------------------------------------------ 3292

AvrL2-A     TTCAAGGTGGAAGATTTGCCTAAGGAAGAAGAACTCGTCAAATATAAAGCTGATGAGGTG 480
sc8713_1    TTCAAGGTGGAAGATTTGCCTAAGGAAGAAGAACTCGTCAAATATAAAGCTGATGAGGTG 309
sc8713_2    ------------------------------------------------------------ 3292

AvrL2-A     CCTCCTCCACGTTATGATGATTATTTCATTAAAACCCCTAAATGA 525
sc8713_1    CCTCCTCCACGTTATGATGATTATTTCATTAAAACCCCTAAATGA 354
sc8713_2    --------------------------------------------- 3292
```

```
AvrL2-B     ATGTTACTAGTGCGGCATATGCCCTTCCTGACACCGTGCTTTCGTGCATCTCAACTTCGA  60
sc4334      ATGTTACTAGTGCGGCATATGCCCTTCCTGACACCGTGCTTTCGTGCATCTCAACTTCGA  11780

AvrL2-B     AGCTTTTGTTTAATTGCTTTCTTATTGTGTCAATCCCTTCAATCCATCGTCTCACTGCCA  120
sc4334      AGCTTTTGTTTAATTGCTTTCTTATTGTGTCAATCCCTTCAATCCATCGTCTCACTGCCA  11720

AvrL2-B     GCACTTCCCTCCAAAGTGGAGTTGTCAGCACCAAGAATTAAGGCACAAGCACGGGTGAAT  180
sc4334      GCACTTCCCTCCAAAGTGGAGTTGTCAGCACCAAGAATTAAGGCACAAGCACGGGTGAAT  11660

AvrL2-B     CAGTTTGTGAGAGAAAACAACCGTCCCCCTCGTCGAAGTGAACTCAATGCCATCCTTCGC  240
sc4334      CAGTTTGTGAGAGAAAACAACCGTCCCCCTCGTCGAAGTGAACTCAATGCCATCCTTCGC  11600

AvrL2-B     CAGGAAAATCTCAAACTGAACGCAGGTAAGCAATAAGACTTATATCACTATCACTATCTG  300
sc4334      CAGGAAAATCTCAAACTGAACGCAGGTAAGCAATAAGACTTATATCACTATCACTATCTG  11540

AvrL2-B     AGCACTGATCCCAATCTTCTTCGACGCGGGTATCCAGGTTATCTTCCCCCGCAATCGTCA  360
sc4334      AGCACTGATCCCAATCTTCTTCGACGCGGGTATCCAGGTTATCTTCCCCCGCAATCGTCA  11480

AvrL2-B     ACGTCGCACGAGGACAATTGGGAAACTCTTGACAAGGAAATTGAGGAATATCGCAATGGC  420
sc4334      ACGTCGCACGAGGACAATTGGGAAACTCTTGACAAGGAAATTGAGGAATATCGCAATGGC  11420

AvrL2-B     AAAAGTTTCAAGGTGGAAGATTTGCCTAAGGAAGAAGAACTCGTCAAATATAAAGCTGAT  480
sc4334      AAAAGTTTCAAGGTGGAAGATTTGCCTAAGGAAGAAGAACTCGTCAAATATAAAGCTGAT  11360

AvrL2-B     GAGGTGCCTCCTCCACGTTATGATGATTATTTCATTAAAACCCCTAAATGA    531
sc4334      GAGGTGCCTCCTCCACGTTATGATGATTATTTCATTAAAACCCCTAAATGA    11309
```

```
AvrL2-C      ATGGGCAAAGGAAATAACATTCAAACGCCGTCCTTTCGTGCATCTCGACTTCGAAGCTTT  60
AvrL2-D      ATGGGCAAAGGAAATAACATTCAAACGCCGTCCTTTCGTGCATCTCGACTTCGAAGCTTT  60
sc275_1      ATGGGCAAAGGAAATAACATTCAAACGCCGTCCTTTCGTGCATCTCGACTTCGAAGCTTT  11646
sc275_2      ATGGGCAAAGGAAATAACATTCAAACGCCGTCCTTTCGTGCATCTCGACTTCGAAGCTTT  16625

AvrL2-C      TGTTTAATCGCTTTCTTATTGTGTCAATCACTTCAATCTATCGTCTCACTACCAAAAATT  120
AvrL2-D      TGTTTAATCGCTTTCTTATTGTGTCAATCACTTCAATCTATCGTCTCACTACCAACAATT  120
sc275_1      TGTTTAATCGCTTTCTTATTGTGTCAATCACTTCAATCTATCGTCTCACTACCAAAAATT  11586
sc275_2      TGTTTAATCGCTTTCTTATTGTGTCAATCACTTCAATCTATCGTCTCACTACCAAAAATT  16565

AvrL2-C      TCCCCTGCTGAGGTGTCAGCATTGAAAACTCGCGTACGAGCACGGTTGGATCAGTTTTGC  180
AvrL2-D      TCCCCTGCTGAGGTGTCAGCATTGAAAACTCGCGTACGAGCACGGTTGGATCAGTTTTGC  180
sc275_1      TCCCCTGCTGAGGTGTCAGCATTGAAAACTCGCGTACGAGCACGGTTGGATCAGTTTTGC  11526
sc275_2      TCCCCTGCTGAGGTGTCAGCATTGAAAACTCGCGTACGAGCACGGTTGGATCAGTTTTGC  16505

AvrL2-C      AGAGAACAAGGCTATAGACCTGAAAACAGTCAATTCTTTGCCATCCTT------------  228
AvrL2-D      AGAGAACAAGGCTATAGACCTGAAGACAGTCAATTCTTTGCCATCGTT------------  228
sc275_1      AGAGAACAAGGCTATAGACCTGAAGACAGTCAATTCTTTGCCATCGTTCNGTACTTTTTG  11466
sc275_2      AGAGAACAAGGCTATAGACCTGAAAACAGTCAATTCTTTGCCATCCTT------------  16457

AvrL2-C      ------------------------------------------------------------  228
AvrL2-D      ------------------------------------------------------------  228
sc275_1      ATATTAGTCTGTATGTTTTTTTCAATATGCCACTTGCAGAGTAGGTGATGTGAATGTGGG  11406
sc275_2      ------------------------------------------------------------  16457

AvrL2-C      ------------------------------------------------------------  228
AvrL2-D      ------------------------------------------------------------  228
sc275_1      AGTACATGATTCAAACTATTCATTAAGGCTAACTCTCGATCGGTCACAACAGCGCTTATC  11346
sc275_2      ------------------------------------------------------------  16457

AvrL2-C      ------------------------CGCGAGGAATATGCGCAGATCAAACCGCAATTTGGA  264
AvrL2-D      ------------------------CGCGAGGAATATGCGCAGATCAAACCGCAATTTGGA  264
sc275_1      TGTAGTGACTTTGAGCCCAAGGANCGCGAGGAATATGCGCAGATCAAACCGCAATTTGGA  11286
sc275_2      ------------------------CGCGAGGAATATGCGCAGATCAAACCGCAATTTGGA  16421

AvrL2-C      CACGCGCAGTACGCTCCAGCACACTCGCGCGACTCAGCTCCAAGTCACCAGGCAGAACAG  324
AvrL2-D      CACGCGCAGTACGCTCCAGCACACTCGCGCGACTCAGCTCCAAGTCACCAGGCAGAACAG  324
sc275_1      CACGCGCAGTACGCTCCAGCACACTCGCGCGACTCAGCTCCAAGTCACCAGGCAGAACAG  11226
sc275_2      CACGCGCAGTACGCTCCAGCACACTCGCGCGACTCAGCTCCAAGTCACCAGGCAGAACAG  16361

AvrL2-C      AATTATGGCACAGGTAAGCAATAAGACTTAAATCACTATCACTATCTGAGCACTAATCCC  384
AvrL2-D      AATTATGGCACAGGTAAGCAATAAGACTTAAATCACTATCACTATCTGAGCACTAATCCC  384
sc275_1      AATTATGGCACAGGTAAGCAATAAGACTTAAATCACTATCACTATCTGAGCACTAATCCC  11166
sc275_2      AATTATGGCACAGGTAAGCAATAAGACTTAAATCACTATCACTATCTGAGCACTAATCCC  16301

AvrL2-C      AATCTTCTTCGACGTGGGTTTCCAGGTGTTTTTCCCTCGCGAGTGACCCCAGGCATTGAA  444
AvrL2-D      AATCTTCTTCGACGTGGGTTTCCAGGTGTTTTTCCCTCGCGAGTGACCCCAGGCATTGAA  444
sc275_1      AATCTTCTTCGACGTGGGTTTCCAGGTGTTTTTCCCTCGCGAGTGACCCCAGGCATTGAA  11106
sc275_2      AATCTTCTTCGACGTGGGTTTCCAGGTGTTTTTCCCTCGCGAGTGACCCCAGGCATTGAA  16241

AvrL2-C      GATTGGGAAAAGAAAATGCACGACCAAGATTGGGAACATTATGAACGTGCCATTGTGGAG  504
AvrL2-D      GATTGGGAAAAAAAAATGCACGACCAAGATTGGGAACATTATGAACGTGCCATTGTGGAG  504
sc275_1      GATTGGGAAAAAAAAATGCACGACCAAGATTGGGAACATTATGAACGTGCCATTGTGGAG  11046
sc275_2      GATTGGGAAAA-------------------------------------------------  16230

AvrL2-C      TTTATGAATGACAAAGGTATTTGGCTGCCTGATATTGATGAGGTCCACGGTATTCCGCCG  564
AvrL2-D      TTTATGAATGACAAAGGTGCTTGGCTGCCTGATATTGATGAGGTCCACGGTATTCCGCCG  564
sc275_1      TTTATGAATGACAAAGGTGCTTGGCTGCCTGATATTGATGAGGTCCACGGTATTCCGCCG  10986
sc275_2      ------------------------------------------------------------  16230

AvrL2-C      CCTGAACCTTATATTTTTTCATCCCAAAAAAATTAG    600
AvrL2-D      CCTGAACCTTATATTTTTTCATCCCAAAAAAATTAG    600
sc275_1      CCTGAACCTTATATTTTTTCATCCCAAAAAAATTAG    10950
sc275_2      ------------------------------------    16230
```

Additional file 15. ***AvrL2* family DNA sequences.**
Alignment of *AvrL2* coding regions with their closest matching scaffolds in the *M. lini* genome assembly. Polymorphic nucleotides are shaded blue.

Note that sc4334 and sc8713 are short scaffolds of 13,347 bp and 3,292 bp, respectively, suggesting that they might be located in a repetitive region of the genome that is difficult to assemble. Also sc8713 contains partial coding regions at the 5’ and 3’ ends of the scaffold that are homologous to the candidate identified on sc4334, which is characteristic of scaffolds formed from collapsed repeats.
