# Supplementary material for: Genome analysis and avirulence gene cloning using a high-density RADseq linkage map of the flax rust fungus, Melampsora lini
Source: BMC Genomics. 2016 Aug 22;17(1):667. doi: 10.1186/s12864-016-3011-9 (PMC4994203; doi:10.1186/s12864-016-3011-9)
Supplement: Additional file 17: — Sequence characteristics in the regions surrounding avirulence genes. Table showing the number of scaffolds, cumulative scaffold length and sequence characteristics of scaffolds that contain and/or contain markers that co-segregate with avirulence genes and the I-1 inhibitor gene. (DOCX 16 kb) [file 12864_2016_3011_MOESM17_ESM.docx]

| Region | LG | Position (cM) | Scaffolds | Total length (kb) | %GC | %N | % repeat | Genes per 100 kb | Notes |
| --- | --- | --- | --- | --- | --- | --- | --- | --- | --- |
| *I-1* | 1 | 8.58 | 1 | 72.9 | 39.60 | 7.88 | 26.76 | 4.12 |  |
| *AvrL11* | 4 | 202.62 / 203.27 | 23 | 657.1 | 40.50 | 11.82 | 24.12 | 6.54 |  |
| *AvrL567* | 4 | 336.88 | 2 | 42.7 | 40.76 | 13.76 | 20.98 | 16.39 | The scaffold that contains *AvrL567* (sc1392) contains a single marker that places it in LG7 but *AvrL567* maps to LG4. This suggests that the scaffold is chimeric |
| *AvrM* | 5 | 281.1 | 2 | 58.5 | 40.43 | 9.05 | 40.82 | 6.84 | All five avirulence paralogues and the virulence allele have assembled as a single gene in sc4666 |
| *AvrN* | 5 | 339.79 / 340.44 / 341.09 | 3 | 52.9 | 38.34 | 44.15 | 14.09 | 7.56 | The three scaffolds that contain markers co-segregating with *AvrN* contain a high percentage of N’s |
| *AvrM3* | 12 | 168.99 | 1 | 90.7 | 40.84 | 12.09 | 16.94 | 14.33 |  |
| *AvrP4* | 13 | 153.01 | 1 | 83.8 | 40.38 | 4.72 | 37.94 | 11.94 |  |
| *AvrP/P123* | 15 | 116.73 / 117.32 | 1 | 83.3 | 42.68 | 17.28 | 19.69 | 9.60 | The scaffold that contains *AvrP123* (sc558) contains five markers that co-segregate with one another and place the scaffold in LG11, but *AvrP* and *AvrP123* map to LG15. This suggests that the scaffold is chimeric |
| *AvrL2* | 16 | 124.47 | 37 | 811.2 | 41.17 | 11.96 | 29.98 | 9.49 | The *AvrL2* locus has significant assembly issues as outlined in this manuscript |
| *AvrM14* | 19 | 1.95 | 2 | 138.6 | 41.05 | 7.60 | 48.45 | 7.21 | The scaffold that contains *AvrM14* (sc27) contains a 171 bp insertion in the region corresponding to the *AvrM14* coding region |
| Genome-wide | n/a | n/a | 21,310 | 189,516.7 | 40.94 | 14.12 | 25.24 | 8.62 |  |

**Additional file 17. Sequence characteristics in regions surrounding avirulence genes.**

Sequence characteristics were determined in the region surrounding each avirulence gene using all scaffolds that contained an avirulence gene, and/or contained markers that co-segregated with an avirulence gene.
