# Supplementary material for: Genome analysis and avirulence gene cloning using a high-density RADseq linkage map of the flax rust fungus, Melampsora lini
Source: BMC Genomics. 2016 Aug 22;17(1):667. doi: 10.1186/s12864-016-3011-9 (PMC4994203; doi:10.1186/s12864-016-3011-9)
Supplement: Additional file 18: — Primers used for PCR amplification of avirulence gene sequences. Table showing primer sequences, amplicon lengths and uses of all primers used to amplify AvrM14 or AvrL2 sequences. (DOCX 20 kb) [file 12864_2016_3011_MOESM18_ESM.docx]

| **Forward primer** | **Sequence (5’-3’)** | **Reverse primer** | **Sequence (5’-3’)** | **Product size** | | **Use(s)** |
| --- | --- | --- | --- | --- | --- | --- |
|  |  |  |  | **Genomic DNA** | **cDNA** |  |
| C34-1 | CACATTGATCCCGTTCATAAC | C34-4 | GATTTTCGGAACAAAGGAATG | 861 bp | n/a | *AvrM14-A* and *AvrM14-B* cloning. *AvrM14* CAPS marker. Positive PCR control for *AvrL2* SCAR markers |
| AvrM14-C1-F4 | AATTCATGGCCGGCAATAATGATCTT | AvrM14-C1-R2 | AATTCTCAGAAAAAGCTGTGTAGTTG | 531 bp | 454 bp | Analysis of *AvrM14-A* and *AvrM14-B* gene expression by RT-PCR. Amplification of *AvrM14-A* and *AvrM14-B* cDNAs to generate plant gene expression constructs that lacked the predicted signal peptide |
| SC275.1a | CTCTTTTCCTCTGCCAACA | ML5 | GAACTCATCGTTCCATTCGC | 4,431 bp | n/a | *AvrL2-A* cloning |
| SC4334.2 | GTCTTAGTACTACATGTAATTG | ML13 | CATGGATACTTGAGAACTGC | 1,068 bp | n/a | *AvrL2-B* cloning |
| SC275.1b | CTCTTTCGCTCTGGCAACC | SC275.3 | TTGATCGATGCAAGACGCTG | 1,032 bp | n/a | *AvrL2-C* cloning |
| SC275.1a | CTCTTTTCCTCTGCCAACA | SC275.3 | TTGATCGATGCAAGACGCTG | 1,032 bp | n/a | *AvrL2-D* cloning |
| AL2-W | ATGGGCAAAGGAAATAACATTC | AL2-R | TCATTTAGGGGTTTTAATGAAA | 525 bp | 453 bp | Analysis of *AvrL2-A* gene expression by RT-PCR. Amplification of *AvrL2-A* cDNA to generate a plant gene expression construct containing the complete open reading frame |
| VL2-W | ATGTTACTAGTGCGGCATATG | AL2-R | TCATTTAGGGGTTTTAATGAAA | 531 bp | 459 bp | Analysis of *AvrL2-B* gene expression by RT-PCR |
| SC275.13 | AACAGTCAATTCTTTGCCATCC | SC275.12 | TCAATATCAGGCAGCCAAAT | 338 bp | 266 bp | Analysis of *AvrL2-C* gene expression by RT-PCR |
| SC275.14 | GACAGTCAATTCTTTGCCATCG | SC275.11 | TCAATATCAGGCAGCCAAGC | 338 bp | 266 bp | Analysis of *AvrL2-D* gene expression by RT-PCR |
| SC4334.1 | GGGCACTCTTTTCCTCTGC | AN255 | GCTAATTTGGACCTAATTCATTA | 733 bp | n/a | *AvrL2-A* SCAR marker |
| SC275.1b | CTCTTTCGCTCTGGCAACC | SC275.12 | TCAATATCAGGCAGCCAAAT | 722 bp | n/a | *AvrL2-C* SCAR marker |
| SC275.1a | CTCTTTTCCTCTGCCAACA | SC275.11 | TCAATATCAGGCAGCCAAGC | 722 bp | n/a | *AvrL2-D* SCAR marker |
| AL2-M | ATGCTGCCAGCACTTTCCTCCAAA | AL2-R | TCATTTAGGGGTTTTAATGAAA | 420 bp | 348 bp | Amplification of *AvrL2-A* cDNA to generate a plant gene expression construct that lacked the predicted signal peptide (dSP36) |
| GW-AvrL2 | caccATGGGCAAAGGAAATAACATTC | AvrL2+stop | TCATTTAGGGGTTTTAATGAAATAATC | 529 bp | 457 bp | Gateway cloning of *AvrL2-A* full-length |
| GW-AvrL2d20 | caccatgTGTTTAATTGCTTTCTTATTGTGTC | AvrL2+stop | TCATTTAGGGGTTTTAATGAAATAATC | 472 bp | 400 bp | Gateway cloning of *AvrL2-A* dSP20 |
| GW-AvrL2d26 | caccatgTTGTGTCAATCCCTTCAATCC | AvrL2+stop | TCATTTAGGGGTTTTAATGAAATAATC | 454 bp | 382 bp | Gateway cloning of *AvrL2-A* dSP26 |
| GW-AvrL2d32 | caccatgTCCATCGTCTCACTGCCAGC | AvrL2+stop | TCATTTAGGGGTTTTAATGAAATAATC | 436 bp | 364 bp | Gateway cloning of *AvrL2-A* dSP32 |

**Additional file 18. Primers used for PCR amplification of avirulence gene sequences.**
